# Supplementary material for: Economic and financial viability of a pig farm in central semi-tropical Mexico: 2022–2026 prospective
Source: PLoS One. 2024 May 9;19(5):e0298897. doi: 10.1371/journal.pone.0298897 (PMC11081355; doi:10.1371/journal.pone.0298897)
Supplement: Attachment — Submitted filename: PONE-D-23-21718_R1_reviewer.pdf [file pone.0298897.s005.pdf]

PLOS ONE

Economic and financial viability of a pig farm in central semi-tropical Mexico: 2022-2026 prospective

--Manuscript Draft--

**Manuscript Number:**	PONE-D-23-21718R1
**Article Type:**	Research Article
**Full Title:**	Economic and financial viability of a pig farm in central semi-tropical Mexico: 2022-2026 prospective
**Short Title:**	Prospective economic and financial outcomes of a pig farm in Mexico
**Corresponding Author:**	Elein Hernandez<br>Universidad Nacional Autonoma de Mexico Facultad de Estudios Superiores Cuautitlán: Universidad Nacional Autonoma de Mexico Facultad de Estudios Superiores Cuautitlan<br>Cuautitlan Izcalli, Estado de Mexico MEXICO
**Keywords:**	Planning horizon; Forecasting; Competitiveness; livestock
**Abstract:**	To estimate the economic and financial viability of a pig farm in central sub-tropical Mexico within a 5-year planning horizon, a Monte Carlo simulation model was utilized. Net returns were projected using simulated values for the distribution of input and product processes, establishing 2021 as base scenario. A stochastic modelling approach was employed to determine the economic and financial outlook. The findings reveal a panorama of economic and financial viability. Net income increased by 555%, return on assets rose from 3.36% in 2022 to 11.34% in 2026, and the probability of decapitalization dropped from 58% to 13%, respectively in the aforesaid periods. Similarly, the probability of obtaining negative net income decreased from 40% in 2022 to 18% in 2026. The technological, productive, and economic management of the production unit allowed for a favourable scenario within the planning horizon. There is a growing interest in predicting the economic sectors worth investing in and supporting, considering their economic and development performance. This research offers both methodological and scientific evidence to demonstrate the feasibility of establishing a planning schedule and validating the suitability of the pork sector for public investment and support.
**Order of Authors:**	Francisco Ernesto Martinez-Castañeda
	Nicolás Callejas-Juárez
	Oscar Cuevas-Reyes
	Nathaniel Alec Rogers-Montoya
	Germán Goméz-Tenorio
	María Elena Trujillo-Ortega
	Claudia Giovanna Peñuelas-Rivas
	Elein Hernandez
**Response to Reviewers:**	According to reviewers' suggestions the following changes were made to the original manuscript:<br>Title page:<br>Lines 3 to 5: Ampersand symbol was included.<br>Line 24: The statement "&These authors also contributed equally to this work" was included.<br>Introduction:<br>Line 41: number "1" was replaced by the word "one".<br>Material and Methods:<br>Line 96 to 98: The following statement was included "An approval for field site access was not required since no field tests were carried out. Only data provided by the pork producers was gathered."<br>Line 143: Table 1 is cell-based.

Powered by Editorial Manager® and ProduXion Manager® from Aries Systems Corporation

	Regarding the reviewer question about the model used: “The authors state they used the probability distributions of Zavala-Pineda et al. (2012) and Posadas-Domínguez et al. The authors must clarify which probability distributions (triangular, Gaussian, etc....) they used and why they were adequate for the case study”<br>Indeed, the probability distributions of Zavala-Pineda et al. and Posadas-Domínguez et al. were used. However, it should be clarified that both latter studies employed an empirical distribution based on the model developed by Richardson, J.W., S.L. Klose, and A.W. Gray. 2000. An applied procedure for estimating and simulating multivariate empirical (MVE) probability distributions in farm level risk assessment and policy analysis. J. Agric. Appl. Econ. 32(2):299-315.<br>The following paragraph was included:<br>Line 110-113: According to [16], Empirical multivariate… …the next five years with 500 iterations using the software [18].<br>Regarding the reviewer question about the package used to perform the quantitative analysis:<br>The package used to make the estimations was SIMETAR©<br>Regarding the reviewer question: “It is not clear what exactly t[illegible]uthors are analyzing. I would expect a representative cash flow (a figure) representing all variables considered”<br>While a figure representing all considered variables was not included in the manuscript, the following paragraphs were included for a better understanding of the Materials and Methods section:<br>Line 114 to 124: The following paragraph was included “In order to estimate the parameters for a Multivariate Empircal (MVE) distribution… …. The seventh step completes the parameter estimation for a MVE distribution.”<br>Line 125 to 135: The following paragraph was included “The complete MVE probability distribution was simulated… … fractional deviates (CFD) and expansion factor (Ei).”<br>Regarding the reviewer question: “What are the necessary investments and costs for the studied farm? What are the empirical data applicable to this case study?”<br>The following statement was included:<br>Line 93 to 94: “All economic and financial… …accounting sheets.”<br>Regarding the reviewer observation about the Stochastic Variables of the Model: “Again, the authors provide a too-short description of the methodological steps. How many simulations were performed? Please provide details and clarify all steps.”<br>The following paragraph was included:<br>Line 151 to 157: “The stochastic values from the probability distributions… … KOVs that are used by decision makers [19, 20].”<br>Results and Discussion<br>Regarding the reviewer question: “What are the contributions and the practical implications of this study? I expect a profound discussion on this matter in this section.”<br>The following paragraphs were included:<br>Line 273 to 279: “Since in the analyzed pig production system… … regions would have social and environmental implications [36].<br>Line 280 to 292: “While [37] demonstrated that small-scale pig farms… … Net Social Value by -0.1% (-$309,050,545 USD)”<br>Acknowledgements<br>Line 254: The statement “The authors wish to thank the pork producer that kindly provided the information for this study, and the research project 6498/2022CIB of the Universidad Autónoma del Estado de México for the financing of this research” was replaced by “The authors wish to thank the pork producer that kindly provided the information for this study”.<br>References<br>Following the journal’s submission guidelines, the citing style was changed to numbers in square brackets (e.g. [1], [2]) and references are listed in order of appearance.<br>The following citations and references are included in the manuscript to support the modifications made in the Materials and Methods section and in the discussion:<br>16. Richardson et al. (2000); 17. Richardson et al. (2006); 18. Richardson et al. (2007); 36. Villavicencio-Guitíerrez et al. (2021); and 37. Barrón-Aguilar et al. (1995).
**Additional Information:**	
**Question**	**Response**
**Financial Disclosure**	-FEMC is awarded author<br>- Grant number: 6498/2022CIB

Powered by Editorial Manager® and ProduXion Manager® from Aries Systems Corporation

<table>
<tr><td>Enter a financial disclosure statement that describes the sources of funding for the work included in this submission. Review the submission guidelines for detailed requirements. View published research articles from PLOS ONE for specific examples.

This statement is required for submission and will appear in the published article if the submission is accepted. Please make sure it is accurate.

Unfunded studies
Enter: The author(s) received no specific funding for this work.

Funded studies
Enter a statement with the following details:
• Initials of the authors who received each award
• Grant numbers awarded to each author
• The full name of each funder
• URL of each funder website
• Did the sponsors or funders play any role in the study design, data collection and analysis, decision to publish, or preparation of the manuscript?
• NO - Include this sentence at the end of your statement: The funders had no role in study design, data collection and analysis, decision to publish, or preparation of the manuscript.
• YES - Specify the role(s) played.

* typeset</td><td>- Funder: Universidad Autónoma del Estado de México
-URL:uaemex.mx
- NO - The funders had no role in study design, data collection and analysis, decision to publish, or preparation of the manuscript.</td></tr>
<tr><td>Competing Interests

Use the instructions below to enter a competing interest statement for this submission. On behalf of all authors, disclose any competing interests that could be perceived to bias this work—acknowledging all financial support and any other relevant financial or non-financial competing interests.

This statement is required for submission and will appear in the published article if the submission is accepted. Please make</td><td>The authors have declared that no competing interests exist.</td></tr>
</table>

Powered by Editorial Manager® and ProduXion Manager® from Aries Systems Corporation

sure it is accurate and that any funding sources listed in your Funding Information later in the submission form are also declared in your Financial Disclosure statement.<br><br>View published research articles from *PLOS ONE* for specific examples.<br><br>**NO authors have competing interests**<br><br>Enter: *The authors have declared that no competing interests exist.*<br><br>**Authors with competing interests**<br><br>Enter competing interest details beginning with this statement:<br><br>*I have read the journal's policy and the authors of this manuscript have the following competing interests: [insert competing interests here]*<br><br>* typeset	
**Ethics Statement**<br><br>Enter an ethics statement for this submission. This statement is required if the study involved:<br><br>• Human participants<br>• Human specimens or tissue<br>• Vertebrate animals or cephalopods<br>• Vertebrate embryos or tissues<br>• Field research<br><br>Write "N/A" if the submission does not require an ethics statement.<br><br>General guidance is provided below. Consult the submission guidelines for detailed instructions. **Make sure that all information entered here is included in the Methods section of the manuscript.**	N/A

**Format for specific study types**<br><br>**Human Subject Research (involving human participants and/or tissue)**<br>• Give the name of the institutional review board or ethics committee that approved the study<br>• Include the approval number and/or a statement indicating approval of this research<br>• Indicate the form of consent obtained (written/oral) or the reason that consent was not obtained (e.g. the data were analyzed anonymously)<br><br>**Animal Research (involving vertebrate animals, embryos or tissues)**<br>• Provide the name of the Institutional Animal Care and Use Committee (IACUC) or other relevant ethics board that reviewed the study protocol, and indicate whether they approved this research or granted a formal waiver of ethical approval<br>• Include an approval number if one was obtained<br>• If the study involved *non-human primates*, add *additional details* about animal welfare and steps taken to ameliorate suffering<br>• If anesthesia, euthanasia, or any kind of animal sacrifice is part of the study, include briefly which substances and/or methods were applied<br><br>**Field Research**<br><br>Include the following details if this study involves the collection of plant, animal, or other materials from a natural setting:<br>• Field permit number<br>• Name of the institution or relevant body that granted permission	
**Data Availability**<br><br>Authors are required to make all data underlying the findings described fully available, without restriction, and from the time of publication. PLOS allows rare exceptions to address legal and ethical concerns. See the PLOS Data Policy and FAQ for detailed information.	No - some restrictions will apply

A Data Availability Statement describing where the data can be found is required at submission. Your answers to this question constitute the Data Availability Statement and **will be published in the article**, if accepted.<br><br>**Important:** Stating 'data available on request from the author' is not sufficient. If your data are only available upon request, select 'No' for the first question and explain your exceptional situation in the text box.<br><br>Do the authors confirm that all data underlying the findings described in their manuscript are fully available without restriction?	
**Describe where the data may be found in full sentences. If you are copying our sample text, replace any instances of XXX with the appropriate details.**<br><br>• If the data are **held or will be held in a public repository**, include URLs, accession numbers or DOIs. If this information will only be available after acceptance, indicate this by ticking the box below. For example: *All XXX files are available from the XXX database (accession number(s) XXX, XXX.*).<br>• If the data are all contained **within the manuscript and/or Supporting Information files**, enter the following: *All relevant data are within the manuscript and its Supporting Information files.*<br>• If neither of these applies but you are able to provide **details of access elsewhere**, with or without limitations, please do so. For example:<br><br>*Data cannot be shared publicly because of [XXX]. Data are available from the XXX Institutional Data Access / Ethics Committee (contact via XXX) for researchers who meet the criteria for access to confidential data.*<br><br>*The data underlying the results presented in the study are available from (include the name of the third party*	All relevant data are within the manuscript and its Supporting Information files.

and contact information or URL).<br>• This text is appropriate if the data are owned by a third party and authors do not have permission to share the data.<br><br>* typeset	
Additional data availability information:	

Manuscript Click here to access/download;Manuscript;Manuscript.docx

Economic and financial viability of a pig farm in central semi-tropical Mexico: 2022-2026 prospective

Francisco Ernesto Martínez-Castañeda[1¶], Nicolás Callejas-Juárez[2¶], Oscar Cuevas-Reyes[1&], Nathaniel Alec Rogers-Montoya[3&], Germán Gómez-Tenorio[4&], María Elena Trujillo-Ortega[5&], Claudia Giovanna Peñuelas-Rivas[6&], Elein Hernandez[7&*]

[1] Universidad Autónoma del Estado de México. Instituto de Ciencias Agropecuarias y Rurales. Toluca. México.

[2] Universidad Autónoma del Estado de Chihuahua. Facultad de Zootecnia y Ecología. Chihuahua. México.

[3] Colegio de Postgraduados. Especialidad en Ganadería. Montecillos. México.

[4] Universidad Autónoma del Estado de México. Centro Universitario Temascaltepec. Temascaltepec de González, México.

[5] Universidad Nacional Autónoma de México. Facultad de Medicina Veterinaria y Zootecnia. Coyoacán, México.

[6] Pipestone. FarmTeam Coordinator. Minnesota, United States of America.

[7] Universidad Nacional Autónoma de México. Department of Clinical Studies and Surgery, Facultad de Estudios Superiores Cuautitlán.

*Corresponding author

E-mail: elein_ht@comunidad.unam.mx

[¶]These authors contributed equally to this work.

[&]These authors also contributed equally to this work.

Abstract

To estimate the economic and financial viability of a pig farm in central sub-tropical Mexico within a 5-year planning horizon, a Monte Carlo simulation model was utilized. Net returns were projected using simulated values for the distribution of input and product processes, establishing 2021 as base scenario. A stochastic modelling approach was employed to determine the economic and financial outlook. The findings reveal a panorama of economic and financial viability. Net income increased by 555%, return on assets rose from 3.36% in 2022 to 11.34% in 2026, and the probability of decapitalization dropped from 58% to 13%, respectively in the aforesaid periods. Similarly, the probability of obtaining negative net income decreased from 40% in 2022 to 18% in 2026. The technological, productive, and economic management of the production unit allowed for a favourable scenario within the planning horizon. There is a growing interest in predicting the economic sectors worth investing in and supporting, considering their economic and development performance. This research offers both methodological and scientific evidence to demonstrate the feasibility of establishing a planning schedule and validating the suitability of the pork sector for public investment and support.

Introduction

Mexico is a country with a supply deficit in pork. According to statistical data, 2021 ended with an import volume (not including skin, brine, or other subproducts) of 969,358,505 kg of pork [1], representing just over 12 million pigs [2]. While food production in Latin America mainly stems from small-scale farms [3], only 30% of these farms make it to a second generation, and only between 10% and 15% make it to a third generation [4]. In developing countries, the livestock sector encompasses almost one billion small-scale livestock production units that contribute with 40% of the agricultural GDP, and between 2% and more than 33% of the household income [5]. Given the complex scenario regarding generational transition in farms, methodical planning and economic and financial forecasting represent important tools for decision-making.

According to official data, medium-scale pig farming (between 81 and 1500 animals) contributes between 20% and 30% of the total pork production in Mexico. Since the opening of trade in the said

country, pig farming has been one of the most affected productive sectors and has lost a large part of its production capacity [6, 7, 2]. The study of [8], concluded that a subsidy to the supply function of pork carcass would improve the benefit to society by increasing production and protecting consumers. Similarly, the latter authors evaluated the application of a 20% *ad valorem* tariff, concluding that the sector is sensitive to such tariff and the current policy protects national producers; however, it has a negative impact on both importers and consumers [9], in a market with a deficit in pork supply. According to [10], pork production is an economic sector that has a total multiplier of 3.40, which strategically positions it as a driving force of the economy. Therefore, it is crucial to assess whether to implement support and transfers to this sector.

A stochastic modelling approach of agricultural systems is a method used to evaluate agricultural management practices considering different risk factors, and to understand how such factors could affect the economic and financial viability, as well as the performance of the said systems in the future [11]. The stochastic model developed by [12], was used to aid vertically integrated pork companies in decision-making and coordination of production, taking into account the uncertainty associated to future price of pork. For their part, [13] used both Monte Carlo simulations and econometric models to assess the economic viability in the long term of Mexican pig farms considering four scenarios: Base scenario; hiring-in labor scenario; purchased inputs scenario: and zero subsidies scenario. Similarly, [14] evaluated the economic and financial viability of small-scale dairy systems in central Mexico. [15] conducted an analysis of competitiveness, profitability, and risk analysis in the cow-calf production system. While both the aforesaid studies demonstrated the importance of carrying out a prospective analysis that included the economic, financial, and political spheres, they were also useful for identifying improvement strategies in the productive and technical activities within the farms. The objective of this study was to evaluate the economic and financial performance of a Representative Pig Production Unit with an average inventory of 150 sows (RPPU150), located in a semi-tropical region of Mexico, considering a planning horizon of 2022-2026.

Materials and methods

RPPU150 description

The studied farm is located in a semi-tropical region of the State of Mexico, with an annual average temperature of 20.3°C, an altitude of 1,100 meters above sea level, a precipitation of 1,158 mm, and coordinates 18°59' and 19°14' north latitude, and 99°49' and 100°14' west longitude.

The production system is a farrow-to-finish operation (piglets that are reared to a weight of 110 kg). Pigs are housed in total confinement and separated according to physiological stage. The cycle begins in the breeding phase with insemination of the sows, once gestation is confirmed, pregnant sows are moved to gestation barns for 116 days, and one week prior to farrowing are transferred to maternity barns for a 23-day lactation period. On average, 12.5 live piglets are born per litter, with a mortality rate of 16%, meaning that 10.5 piglets are weaned at the end of the phase.

Piglets are weaned with an average weight of 7.37 kg, then transferred to nursery barns to consume pre-starter feed for around 26 days until they reach an average weight of 19.28 kg. Subsequently, the developing pigs are moved to a starting area to receive a starter diet for 26 days until they reach an average weight of 35.45 kg. Ultimately, pigs are transferred to fattening barns for 78 days: during the initial 26 days, pigs are fed with a growing diet until they reach a weight of 55.78 kg. Over the subsequent 26 days, they are fed a developing diet until a weight of 80.27 kg is reached. Lastly, pigs are provided a finishing diet for another 26 days until they reach market weight of 110 kg. All economic and financial data were obtained directly from the evaluated farm accounting sheets. An approval for field site access was not required since no field tests were carried out. Only data provided by the pork producers was gathered.

Ethics statement

The Ethics Committee of the Universidad Autónoma del Estado de México (México) approved the research protocol and information gathering tools. Prior to participation, the owner of the farm that conformed the analyzed RPPU150 was provided information about the overall aim and expected outcome of the present research. Due to the nature of the study, no animals were used to obtain data.

The model

A Stochastic simulation approach was carried out to evaluate the economic and financial viability of a representative medium-scale pig farm, using an empiric distribution [16], used by [13, 14] to evaluate Mexican pig farms and small-scale dairy systems, respectively. Historical data for the probability distributions were taken from the [17] database, that comprised 42 years of information (1981–2022). The studied model included data pertaining to the Mexican government to represent the national macroeconomic variables, while data from the International Monetary Fund (IMF) and the United States Department of Agriculture (USDA) were used for the international macroeconomic variables.

According to [16], Empirical multivariate probability distributions (MVE) were estimated for each variable of the analyzed production unit. Historical data from the past ten years were used to estimate the MVEs. The MVEs were then used to simulate stochastic projections of the variables for the next five years with 500 iterations using the software [18].

In order to estimate the parameters for a Multivariate Empircal ([illegible]E) distribution, the following steps were conducted: 1) Random and non-random components estimation for each stochastic variable (X ̂_it=a ̂+b ̂*Trend+c ̂Z_t) were separated; 2) The random component of each stochastic variable (e ̂_it=X_it-X ̂_it). were calculated; 3) Residuals (e ̂_it) were converted to relative deviates about their respective deterministic components; 4) The relative deviates (D_it=e ̂_it/X ̂_it) were sorted and pseudo-minimums and pseudo-maximums created for each random variable; 5) A probability of $P_{min} = Minimum\ S_{it} * 1.000001$ to $P_{max} = maximum * S_{it} * 1.000001$ with $S_{it} = Sorted[D_{it} from \min to\ max]$was assigned to each of the sorted deviates; 6) Calculate the M x M intra-temporal correlation matrix for the M random variables; and 7) The intertemporal correlation coefficients were calculated for the random variables. The seventh step completes the parameter estimation for a MVE distribution.

The complete MVE probability distribution was simulated in excel using Risk. T[illegible]llowing steps were carried out: 1) A sample of Independent Standard Normal Deviates (ISND) was generated. ISND=Risknormal(0,1); 2) To simulate a MVE distribution the ISNDs within each year of the simulation period (k = 1, 2, ... , K) were correlated by multiplying the factored correlation matrix (R_{ij}) and eight of the values in the ISND vector (CSND_i=R_ij*ISND_i); 3) The inter-temporal correlation of the random variables was captured; 4) Equation Adjusted Correlated Standard Normal Deviates (ACSNDs) were

transformed to Correlated Uniform Deviates (CUD). CUD_i=normsdist(ACSND_i); 5) CUD_is were used to simulate random deviates for the empirical distribution of each variable X_i; and 6) The correlated fractional deviates were applied to their respective projected means making any needed adjustments for heteroscedasticity. $\tilde{X}_i = \hat{X}_i * (1 + CFD_i * E_i$, with correlated fractional deviates (CFD) and expansion factor (E_i).

The model projected economic and financial viability and provided information on trends for macroeconomic variables. In order to reduce estimation errors, trends in regional variables were estimated for the period of 2022-2026. The model operates annually at a strategic level and generates financial pro-forma reports that contain results used to calculate key output variables such as net incomes in cash, final cash reserves, change in net real capital, net present value (NPV) and rate of return over assets (RRA), which can inform decision making at both the farm and policy levels. The said financial reports are generated from functional equations that link pig production, sales, production and purchase of inputs, capital operations, and consumption and financing operations.

Stochastic variables of the model

The inputs prices and yield of crops in the RPPU150 were obtained via meetings with producers and direct interviews. A Multivariate Empirical Distribution (MVE) was used to simulate the cost of crops and pigs. The probability distribution parameters were estimated with the Latin hypercube method, used as a sampling procedure for the simulation of pseudo-random numbers.

The use of such method ensures that the coefficient of variation (CV) and the mean for the simulated random variables are equal to the CV and the mean for the historic variables.

The stochastic values from the probability distributions are used in accounting equations to calculate production, receipts, costs, cash flows, and balance sheet variables for the project. Stochastic values sampled from the probability distributions make the financial statement variables stochastic, which are iterated 500 times using random values for the risky variables, the obtained information is used to estimate empirical probability distributions for the Key Output Variables (KOVs) such as present value of

ending net worth, net present value, annual cash flows, amongst others. Providing forecasted KOVs that are used by decision makers [19, 20].

Economic and financial viability indicators

The analysed indicators were total income (TI), total costs (TC), net income in cash (NIC), reserves in cash (RIC), NPV, RRA and cost to benefit ratio (C/B).

Model assumptions

The analysis took into account the following assumptions: (1) scale of production; (2) productivity; (3) farm infrastructure capacity and utilized; (4) technical coefficients are held constant during the planning horizon 2022-2026; (5) the technological level was also held unaltered; and (6) discount rate was established at 12%.

Economic values used were transformed in USD with a currency rate of 19.4143:1 USD:MXN according to Banco de Mexico (December 30, 2022).

Results and discussion

Table 1 shows the economic viability with a noticeable increase in all accounting entries. Gross income grew by 27.93% within the planning horizon, while expenses had a 16.09% reduction, this allowed for a 36% average annual growth of net income. The probability of falling into negative indicators was considerably reduced, with the cash flow deficit dropping from 40% in 2022 to 18% in 2026; the probability of refinancing decreased by 16 percentage points, and the probability of a return on assets less than zero dropped from 58% in 2022 to 13% in 2026.

Table 1. Economic viability of a RPPU150 (Thousands USD).

	2021	2022	2023	2024	2025	2026
Gross income	869.05	911.74	960.46	1,015.28	1,058.45	1,111.82

Total expenditure	849.98	889.93	903.38	926.69	958.39	986.76
Net income	19.07	21.81	57.08	88.59	100.07	125.06
Return on assets (%)	3.36	1.50	6.59	10.21	10.24	11.34
P(Cash Flow deficit) (%)	-	40.00	34.00	24.80	24.00	18.00
P(Refinancing) (%)	-	20.60	17.80	10.80	6.00	4.60
P(Return on assets < 0) (%)	-	58.00	46.00	32.20	21.20	13.00

P=Probability.

Net cash income performance within the planning horizon, according to the risk evaluation, showed that it is possible to fall in bankruptcy (5th percentile). However, 2026 economic panorama is a viable one (Fig 1).

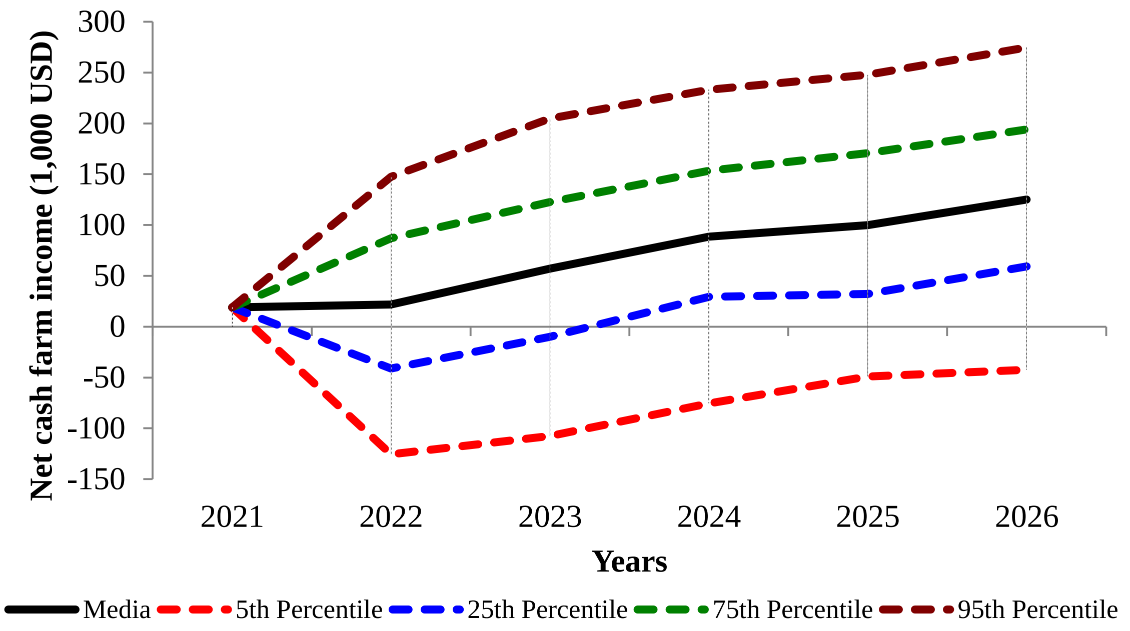

Figure 1. Net cash farm income performance according to percentile and risk simulation.

The cost structure was mainly composed of feed (88.17% of total), followed by labor costs (5.79%), breeding costs (3.46%), and fuel costs (1.26%). Other concepts such as maintenance, veterinary

service expenses, indirect expenses, transportation, and land costs, accounted for 0.79%, 0.47%, 0.03%, 0.02%, and 0.005% of the total costs, respectively.

In the evaluated RPPU150, there is a null probability of obtaining a negative Net Present Value (NPV). The NPV in 2026 finished in 46.9964 thousand USD. The Internal Rate of Return (IRR) was 11.06%. The Cost/Benefit ratio was 0.98 and the Real Rate of Return was 0.07%.

Around 1.7 million tons (MT) of pork were produced in Mexico in 2021 [17], and the State of Mexico contributed with just over 22 thousand tons. Specifically, the said country had an average annual growth rate of 0.74, while the State of Mexico had a growth rate of -3.32 from 1980 to 2021.

In Mexico, the pig farming sector can be described in three historical and conjunctural periods [21]: an initial period of growth driven by the structural policies established in the second half of the 1970s; a second period of crisis in the sector that began in 1985 and ended in 1991; and a third period starting from 1991 with a growing trend until 2021, mainly driven by the consolidation of corporate pig farming systems.

The macroeconomic and sectoral policies adopted by Mexico in the last two decades has led to a change in the production structure of pig farming at the national and local level. While the State of Mexico is in the Central East region of Mexico, where growth and dynamism had positive effects during 1994 to 2012, the average annual growth rate for the said state was -1.31%.

One of the main effects observed in the Mexican pig industry was the impact of trade liberalization on the sector, mainly due to the competitive advantages of the United States over Mexico [22]. The difference between harm to producers and the benefit to consumers, namely $14.0 billion USD, remained in the hands of pork importers and traders, as in Mexico, among other causes, an asymmetric price transmission exists due to an oligopolistic market where retailers tend to quickly pass on price increases to consumers, while delaying the decrease in prices when they have decreased for the producer [23]. Such is the dependence on the external market that a 15% and 21% currency depreciation of the Mexican Peso would only imply an increase in national production of 0.3% and 0.4%, respectively [24].

The transition in the pig farming structure in the State of Mexico is mainly derived from technological progress, defined as feed conversion, and to a lesser extent, the price of feed and the price of pork, with average elasticity values of 1.174, -0.039, and -0.099, respectively [25].

In this regard, [26] determined that the best profit for pig farmers was obtained by producing animals with a range in live weight between 66 and 162 kg. However, the production of heavier carcasses did not necessarily imply a higher profit [25]. The latter authors also found that the Technical Optimum Level (TOL) and Economic Optimum Level (EOL) for pork carcass weight were 94.47 kg and 90.96 kg, respectively, with profits of USD$ 162.1 and USD$ 173.0. In the case of secondary cuts, TOL and EOL were obtained with respective carcass weights of 85.4 kg and 85.3 kg, with profits of USD$ 236.7 and USD$ 236.9, while for tertiary cuts, 82.38 kg (TOL) and 82.26 kg (EOL) were equivalent to profits of USD$ 217.6 and USD$ 217.9.

The State of Mexico used to be one of the main pork producing states. Unlike national pork production, that recovered from 1991, the aforesaid state's pork industry was not able to do so, mainly due to the closure of a large part of its production units. While pork production at the national level grew an average annual rate of 2.63% from 1990 to 2021, it decreased by 1.20% in the State of Mexico. The observed trend in the production volume of pork in Mexico support global projections that estimate an increase of pork mainly in low and middle-income countries [27], but the outlook is not the same for the pork industry in the State of Mexico. In this context, the estimation of financial projections has become a useful tool for assessing economic and financial viability of livestock systems. However, models must consider the conditions which prevail in the studied regions in order to reduce variability [28].

While the State of Mexico is a large consumer of pork, much of the said product is not produced in the state. One of the most significant changes from 2000 to 2019 was the concentration and commercialization of 21% of the slaughterhouses within the state. Additionally, 17 distribution centres and 24 slaughterhouses were located in the Central region of Mexico, representing 90% of the national traded volume [29].

Since 2006, a significant increase in the analysis of data pertaining to the pork sector has driven the development of models to assist decision-making [30]. The focus of these models is mainly related to diseases, DNA analysis, and feeding strategies.

Several studies have been conducted to measure the competitiveness of the pork industry at both the national and State levels in Mexico. By the end of the 2000s, [6] reported favorable private profitability indicators in small, medium, and large-scale pig farms in the State of Mexico. Meanwhile, [7] reported private gains in the sale of weaned piglets in small-scale pig farms (5 to 100 breeding sows). However, none of the aforesaid studies included a risk analysis component like the present study.

In Brazil, [31] applied a stochastic model in a typical full-cycle farm (1500 sows) to evaluate the impact on private and social profits of using locally produced co-products in finishing diets, finding a high coefficient of variation (75-87%) in profits due to volatility in prices and variations in pig biological parameters. These results demonstrate the importance of considering risk factors in strategic planning for livestock businesses.

On the other hand, [32] generated a predictive model to evaluate the effect of making changes in the production system (increasing housing area to reach a higher market weight vs. feeding finishing feed at an earlier age), finding that the modification of feeding systems is the best alternative to increase farm income with a reduced investment. In our study, feeding represented 88% of the total cost structure.

The models of [26, 32, 33] allow for a clear visualization, in economic and biological terms, which option offers greater productivity. [34] reported that optimizing strategies related to feeding and distribution can increase gross income per pig by up to 10%. All the aforesaid models developed to estimate profitability do not consider micro and macroeconomic variables, and are focused on biological components, as well as the price of some inputs for livestock feed.

The results of [35] warn that with the increase in the price of feed and land, the advantages of large-scale pig farms gradually become prominent, thus, forcing small and medium-scale farms to close. Additionally, since pig farming is inevitably linked with the environment and productivity levels, environmental legislation could have a strong impact on the future development of the pork sector [27].

[33] highlight the importance of individual farm modelling, since the combination of curves with different risk factors, production conditions, and payment curves in the same projection, could result in an underestimation of the economic and financial indicators.

Under the current technological, productive and management conditions, considering the macroeconomic and microeconomic outlook presented over the period 2022 to 2026, the RPPU150 showed favorable economic and financial indicators. While the studied scenario was favorable in its mean value, the results showed that there is a risk (5th percentile) of obtaining negative net cash income. Therefore, it is necessary to further analyze the farm's management of production and economic variables. A new prospective study is required given that the present analysis was conducted with a 12% interest rate, making the production unit highly vulnerable in financial terms. According to OECD estimations, a significant pressure on livestock input prices will predominate in the 2020-2030 decade [27].

Since in the analyzed pig production system, 88% of the cost structure was composed of feed, it is important to develop strategies in collaboration with regional agricultural producers that generate benefits for both economic sectors. The goal is not only to ensure livestock feed inputs but also to create short marketing circuits that boost the local economy. While intensive pig production allows for the acquisition of a large volume of inputs, for a production unit with 150 sows (RPPU150), such economies of scale are not an option. Additionally, the acquisition of inputs from distant regions would have social and environmental implications [36].

While [37] demonstrated that small-scale pig farms (100 to 300 sows) were not competitive at private prices, profits at efficient prices increased in comparison to private prices, presenting a comparative advantage in international markets and contributing to 55% of the currency savings for the country that produced the pigs. However, it is important to consider the proper management of the macroeconomic policy related to the Mexican peso/US dollar exchange rate and interest rate since it may impose an additional tax per kilogram of live weight pig produced in Mexico. In this regard, [8] mentioned that a subsidy to the supply of pork would increase production, domestic consumption, and imports by 0.07%, 0.006%, and 0.003%, respectively. The Net Social Value would increase by 0.006%. Since Mexico is an importer of pork, achieving efficiency and competitiveness in this sector is crucial. [9] emphasize the role of possible import tariffs on pork as a protective measure for the sector. The latter authors report that a 20% tariff would increase domestic production by 0.4% (4,774 tons), reduce imports

by 18.9% (19,141 tons), decrease consumption by 0.06% (14,367 tons), and reduce the Net Social Value by -0.1% (-$309,050,545 USD)

Conclusions

The present results evidenced a consistent economic and financial viability scenario for the evaluated pig farm, under the current conditions, and within the analyzed planning horizon. Net income increased by 555%, return on assets rose from 3.36% in 2022 to 11.34% in 2026, and the probability of decapitalization dropped from 58% to 13%, respectively, in the aforesaid periods. Similarly, the probability of obtaining negative net income decreased from 40% in 2022 to 18% in 2026. The technological, productive, and economic management of the production unit allowed for a favorable scenario within the planning horizon. Even with the general favorable scenario, a risk (5th percentile) exists of falling into bankruptcy. It is important to determine the economic and financial performance within a planning horizon, since the analyzed system represents a typical small-scale pig farm in the State of Mexico, which represents 30% of the total pig inventory and 30% of pork production.

Acknowledgements

The authors wish to thank the pork producer that kindly provided the information for this study.

References

1. SIAVI. 2023. Comercio Exterior. Gobierno de México. http://www.economia-snci.gob.mx/ Accessed: May 20, 2022.

2. Martínez Castañeda FE, Ávila Pérez F, Trujillo Ortega ME, Peñuelas Rivas CG. El hecho de nacer en una pocilga, no te da derecho de ser un cerdo. In: Martínez Castañeda, F.E., Herrera Tapia F. (eds) 2020. Aprendizajes y trayectorias del sector agroalimentario mexicano durante el TLCAN. ISBN: 978-92-9248-885-7. Instituto Interamericano de Cooperación para la Agricultura (IICA). Pp. 163-178

3. FAO. Portal lácteo. https://www.fao.org/dairy-production-products/production/production-systems/es/. Accessed January 13, 2022.

4. Camacho Castellanos CM, Vega González M. Primeros pasos para planificar la transición generacional en una granja que es una empresa familiar. https://www.3tres3.com/latam/articulos/primeros-pasos-para-planificar-transicion-generacional-en-una-granja_14944/#:~:text=Seg%C3%BAn%20la%20FAO%20m%C3%A1s%20del,y%2015%25%20a%20la%20tercera. Consulted March 21, 2023. Acceced: November 12, 2023.

5. Rota A. Desarrollo de la ganadería en pequeña escala. Fondo Internacional de Desarrollo Agrícola. 2016. https://www.ifad.org/documents/38714170/40237450/Scaling+up+Results+in+Smallholder+Livestock+Development_s.pdf/f7a33c96-ade3-4ce4-90c3-62431cada563 Accessed 30 March 2022

6. Hernández-Martínez J, Rebollar-Rebollar S, Rojo-Rubio R, García-Salazar JA, Guzmán-Soria E, Martínez-Tinajero JJ, Díaz-Carreño MA. Private profitability of swine farms in the South of the State of Mexico. Universidad y Ciencia. 2008;24:117-124.

7. Bobadilla-Soto EE, Espinoza-Ortega A, Martínez-Castañeda FE. Swine piglet breeding farms competitiveness and profitability. Revista Mexicana de Ciencias Pecuarias. 2013;4:87-92.

8. Rebollar-Rebollar S, Velázquez HH, Gómez-Tenorio G, Posadas-Domínguez RR, Martínez-Castañeda FE. Efectos de la aplicación de subsidios al mercado porcino en México. Archivos de Zootecnia. 2020a;69:30-37. https://doi.org/10.21071/az.v69i265.5036

9. Rebollar-Rebollar S, Gómez-Tenorio G, Martínez-Castañeda FE. Efectos de un arancel Ad Valorem sobre importaciones de carne de cerdo en México. Agricultura, Sociedad y Desarrollo. 2020b;17:697-712. https://doi.org/10.22231/asyd.v17i4.1400

10. Sosa Urrutia ME, Martínez Castañeda FE. Espinosa García JA, Buendía Rodríguez G. Contribución del sector pecuario a la economía Mexicana. Un análisis desde la Matriz Insumo Producto. Revista Mexicana de Ciencias Pecuarias. 2017;8:31-41. https://doi.org/10.22319/rmcp.v8i1.4308

11. Jonasson E, Filipski M, Brooks J, Taylord EJ. Modeling the welfare impacts of agricultural policies in developing countries. Journal of Policy Modeling. 2014;36:63–82. https://doi.org/10.1016/j.jpolmod.2013.10.002

12. Nadal-Roig E, Lluís M, Plà-Aragonès LM, Pagès-Bernaus A, Albornoz VM. A two-stage stochastic model for pig production planning in vertically integrated production systems. Computers and Electronics in Agriculture. 2020:176:105615. https://doi.org/10.1016/j.compag.2020.105615

13. Zavala-Pineda MJ, Salas-González JM, Leos-Rodríguez JA, Sagarnaga-Villegas LM. The building of representative hog farm production and analysis of its economic viability during the period 2009-2018. Agrociencia. 2012;46:731-743.

14. Posadas-Domínguez RR, Callejas-Juárez N, Arriaga-Jordán CM, Martínez-Castañeda FE. Economic and financial viability of small-scale dairy systemsin central Mexico: economic scenario 2010–2018. Tropical Animal Health and Production. 2016;48:1667-1671. https://doi.org/10.1007/s11250-016-1141-3

15. Callejas Juárez N, Rebollar Rebollar S. Rentabilidad y competitividad del sistema vaca-becerro en México. In: Martínez Castañeda FE, Herrera Tapia F. (eds) 2020. Aprendizajes y trayectorias del sector agroalimentario mexicano durante el TLCAN. ISBN: 978-92-9248-885-7. Instituto Interamericano de Cooperación para la Agricultura (IICA). Pp. 121-140.

16. Richardson, J.W., S.L. Klose, and A.W. Gray. 2000. An applied procedure for estimating and simulating multivariate empirical (MVE) probability distributions in farm level risk assessment and policy analysis. J. Agric. Appl. Econ. 32(2):299-315.

17. SIACON. 2022. Sistema de Información agroalimentaria de Consulta. Secretaria de Agricultura y Desarrollo Rural. México. Database.

18. Simetar (2008), Simulation and Econometrics to Analyze Risk, Department of Agricultural Economics, Texas A&M University, College Station, TX.

19. Richardson, J. W. (2006). “Simulation for applied risk management.” Unnumbered staff report, Department of Agricultural Economics, Agricultural and Food Policy Center, Texas A&M University, College Station, Texas.

20. Richardson, J.W., Herbst, B.K., Outlaw, and Chope Hill II, R. 2007. Including Risk in Economic Feasibility Analyses: The Case of Ethanol Production in Texas. Journal of Agribusiness 25,2(Fall 2007):115S132

21. Bobadilla-Soto EE, Espinoza-Ortega A, Martínez-Castañeda FE. Dinámica de la producción porcina en México de 1980 a 2008. Revista Mexicana de Ciencias Pecuarias. 2010;1;251-268.

22. Tinoco JJL. La Porcicultura mexicana y el Tratado de Libre Comercio de América del Norte. Universidad Nacional Autónoma de México. 2004.

23. Gómez-Tenorio G, Rebollar-Rebollar S, Velázquez-Villalva HH. Impacto económico del libre comercio de carne de cerdo en la porcicultura mexicana. In: Martínez Castañeda, F.E., Herrera Tapia F. (eds) 2020. Aprendizajes y trayectorias del sector agroalimentario mexicano durante el TLCAN. ISBN: 978-92-9248-885-7. Instituto Interamericano de Cooperación para la Agricultura (IICA). Pp. 107-119.

24. Almazan-Figueroa O, Rebollar-Rebollar S, Velázquez-Villalva HH, Gómez-Tenorio G, Hernández-Martínez J. Effects of Mexican peso depreciation on pork market. Agronomía Mesoamericana. 2018;29:557-569. https://doi:10.15517/ma.v29i3.30105

25. Rebollar Rebollar A, Gómez Tenorio G, Hernández Martínez J, Rebollar-Rebollar S, González Razo FJ. Behaviour of supply and demand of regional pig carcasses in Mexico. Revista Mexicana de Ciencias Pecuarias. 2014;5:377-392.

26. Rebollar Rebollar S, Gómez Tenorio G, Hernández Matínez J, Rojo Rubio R, González Razo FJ, Avilés Nova F. Determinación del óptimo técnico y económico en una granja porcícola en Temascaltepec, Estado de México. Ciencia Ergo Sum. 2007;14:255-262.

27. OECD/FAO, OECD-FAO Agricultural Outlook 2022-2031, OECD Publishing, Paris, 2022.

28. Zhang F, Wang F. Prediction of pork supply via the calculation of pig population based on population prediction model. International Journal of Agricultural and Biological Engineering. 2020;13:208–217. https://doi.org/10.25165/j.ijabe.20201302.5303

29. Callejas-Juárez N, Martínez-Castañeda FE, Rebollar-Rebollar S. Estructura de mercado para cerdos vivos en México. REDES, Revista Hispana para el Análisis de Redes Sociales. 2020;31:116-123. https://doi.org/10.5565/rev/redes.888

30. van Klompenburg T, Kassahun A. (2022). Data-driven decision making in pig farming: A review of the literature. Livestock Science. 2022:261:104961. https://doi.org/10.1016/j.livsci.2022.104961

31. Ali BM, Berentsen PBM, Bastiaansen JWM, Oude Lansink, A. A stochastic bio-economic pig farm model to assess the impact of innovations on farm performance. Animal. 2018;12:819–830. https://doi.org/10.1017/S1751731117002531

32. Calderón Díaz JA, Shalloo L, Niemi JK, Kyriazakis I, McKeon M, McCutcheon G, Manzanilla EG. Description, evaluation, and validation of the Teagasc Pig Production Model. Journal of Animal Science. 2019;97:2803–2821. https://doi.org/10.1093/jas/skz163

33. Leen F, Van den Broeke A, Aluwé M, Lauwers L, Millet S, Van Meensel J. (2018). Stakeholder-driven modelling the impact of animal profile and market conditions on optimal delivery weight in growing-finishing pig production. Agricultural Systems. 2018;162:34–45.

34. Davoudkhani M, Mahé F, Dourmad JY, Gohin A, Darrigrand E, Garcia-Launay F. Economic optimization of feeding and shipping strategies in pig-fattening using an individual-based model. Agricultural Systems. 2020;184:102899. https://doi.org/10.1016/j.agsy.2020.102899

35. Jia Y, Zhang Z, He Z, Zh, P, Zhang Y, Sun T. Production Efficiency Prediction of Pig Breeding Industry by Optimized LSTM Computer Algorithm under Environmental Regulation. Scientific Programming. 2021;1-12. https://doi.org/10.1155/2021/3074167

https://doi.org/10.1016/j.agsy.2018.01.013

36. Villavicencio- Gutiérrez MR, Rogers- Montoya NA, Martínez- Campos AR, Gómez- Tenorio G, Martínez- Castañeda FE. The environmental performance of different pork production scenarios: a life cycle assessment study. Tropical Animal Health and Production. 2021;54:44 https://doi.org/10.1007/s11250-022-03045-6

37. Barrón-Aguilar JF, García-Mata R, Mora-Flores JS, López-Díaz S, Pró-Martínez, García-Sánchez R. 2000. Competitiveness and the effect of economic policy on hog production in 13 farms in the state of Michoacán, 1995. Agrociencia. 34:369-377.

Click here to access/download
Supporting Information
Data EM150 PlosOne.xlsx

Economic and financial viability of a pig farm in central semi-tropical Mexico: 2022-2026 prospective

Francisco Ernesto Martínez-Castañeda[1¶], Nicolás Callejas-Juárez[2¶], Oscar Cuevas-Reyes[1&], Nathaniel Alec Rogers-Montoya[3&], Germán Gómez-Tenorio[4&], María Elena Trujillo-Ortega[5&], Claudia Giovanna Peñuelas-Rivas[6&], Elein Hernandez[7&*]

[1] Universidad Autónoma del Estado de México. Instituto de Ciencias Agropecuarias y Rurales. Toluca. México.

[2] Universidad Autónoma del Estado de Chihuahua. Facultad de Zootecnia y Ecología. Chihuahua. México.

[3] Colegio de Postgraduados. Especialidad en Ganadería. Montecillos. México.

[4] Universidad Autónoma del Estado de México. Centro Universitario Temascaltepec. Temascaltepec de González, México.

[5] Universidad Nacional Autónoma de México. Facultad de Medicina Veterinaria y Zootecnia. Coyoacán, México.

[6] Pipestone. FarmTeam Coordinator. Minnesota, United States of America.

[7] Universidad Nacional Autónoma de México. Department of Clinical Studies and Surgery, Facultad de Estudios Superiores Cuautitlán.

*Corresponding author

E-mail: elein_ht@comunidad.unam.mx

[¶]These authors contributed equally to this work.

[&]These authors also contributed equally to this work.

Formatted: English (United States)

Formatted: English (United States)

Formatted: English (United States)

Formatted: Font: Not Bold, Superscript

Formatted: Font: Not Bold

Formatted: Font: Not Bold

Abstract

To estimate the economic and financial viability of a pig farm in central sub-tropical Mexico within a 5-year planning horizon, a Monte Carlo simulation model was utilized. Net returns were projected using simulated values for the distribution of input and product processes, establishing 2021 as base scenario. A stochastic modelling approach was employed to determine the economic and financial outlook. The findings reveal a panorama of economic and financial viability. Net income increased by 555%, return on assets rose from 3.36% in 2022 to 11.34% in 2026, and the probability of decapitalization dropped from 58% to 13%, respectively in the aforesaid periods. Similarly, the probability of obtaining negative net income decreased from 40% in 2022 to 18% in 2026. The technological, productive, and economic management of the production unit allowed for a favourable scenario within the planning horizon. There is a growing interest in predicting the economic sectors worth investing in and supporting, considering their economic and development performance. This research offers both methodological and scientific evidence to demonstrate the feasibility of establishing a planning schedule and validating the suitability of the pork sector for public investment and support.

Introduction

Mexico is a country with a supply deficit in pork. According to statistical data, 2021 ended with an import volume (not including skin, brine, or other subproducts) of 969,358,505 kg of pork [1] ~~(SIAVI, 2023)~~, representing just over 12 million pigs [2] ~~(Martinez et al., 2020)~~. While food production in Latin America mainly stems from small-scale farms [3~~(FAO, 2022)~~], only 30% of these farms make it to a second generation, and only between 10% and 15% make it to a third generation [4]~~(Camacho and Vega 2023)~~. In developing countries, the livestock sector encompasses almost one~~1~~ billion small-scale livestock production units that contribute with 40% of the agricultural GDP, and between 2% and more than 33%

of the household income [5]~~(Rota, 2016).~~ . Given the complex scenario regarding generational transition in farms, ~~methodic~~methodical planning and economic and financial forecasting represent important tools for decision-making.

According to official data, medium-scale pig farming (between 81 and 1500 animals) contributes between 20% and 30% of the total pork production in Mexico. Since the opening of trade in the said country, pig farming has been one of the most affected productive sectors and has lost a large part of its production capacity [6, 7, 2]~~(Hernández-Martínez et al., 2008; Bobadilla-Soto et al., 2013; Martínez et al., 2020)~~. The study of [8], ~~Rebollar-Rebollar et al. (2020a)~~ concluded that a subsidy to the supply function of pork carcass would improve the benefit to society by increasing production and protecting consumers. Similarly, the latter authors evaluated the application of a 20% *ad valorem* tariff, concluding that the sector is sensitive to such tariff and the current policy protects national producers; however, it has a negative impact on both importers and consumers [9] ~~(Rebollar-Rebollar et al., 2020b)~~, in a market with a deficit in pork supply. According to [10]~~Sosa et al. (2017)~~, pork production is an economic sector that has a total multiplier of 3.40, which strategically positions it as a driving force of the economy. Therefore, it is crucial to assess whether to implement support and transfers to this sector.

A stochastic modelling approach of agricultural systems is a method used to evaluate agricultural management practices considering different risk factors, and to understand how such factors could affect the economic and financial viability, as well as the performance of the said systems in the future [11]~~(Jonasson et al. 2014)~~. The stochastic model developed by [12]~~Nadal-Roig et al. (2020)~~, was used to aid vertically integrated pork companies in decision-making and coordination of production, taking into account the uncertainty associated to future price of pork. For their part, [13]~~Zavala-Pineda et al. (2012)~~ used both Monte Carlo simulations and econometric models to assess the economic viability in the long term of Mexican pig farms considering four scenarios: Base scenario; hiring-in ~~labour~~labor scenario; purchased inputs scenario: and zero subsidies scenario. Similarly, [14]~~Posadas-Domínguez et al. (2016)~~ evaluated the economic and financial viability of small-scale dairy systems in central Mexico. [15]~~Callejas-Juárez and Rebollar-Rebollar (2020)~~ conducted an analysis of competitiveness, profitability, and risk analysis in the cow-calf production system. While both the aforesaid studies demonstrated the importance of carrying out a prospective analysis that included the economic, financial, and political

Formatted: English (United States)

Formatted: English (United States)

Formatted: Indent: First line: 0.49"

Formatted: English (United States)

Formatted: English (United States)

Formatted: English (United States)

Formatted: English (United States)

Formatted: English (United States)

Formatted: English (United States)

Formatted: English (United States)

Formatted: English (United States)

Formatted: English (United States)

Formatted: English (United States)

Formatted: English (United States)

Formatted: English (United States)

spheres, they were also useful for identifying improvement strategies in the productive and technical activities within the farms. The objective of this study was to evaluate the economic and financial performance of a Representative Pig Production Unit with an average inventory of 150 sows (RPPU150), located in a semi-tropical region of Mexico, considering a planning horizon of 2022-2026.

Materials and methods

RPPU150 description

The studied farm is located in a semi-tropical region of the State of Mexico, with an annual average temperature of 20.3°C, an altitude of 1,100 meters above sea level, a precipitation of 1,158 mm, and coordinates 18°59' and 19°14' north latitude, and 99°49' and 100°14' west longitude.

Formatted: Indent: First line: 0.49"

The production system is a farrow-to-finish operation (piglets that are reared to a weight of 110 kg). Pigs are housed in total confinement and separated according to physiological stage. The cycle begins in the breeding phase with insemination of the sows, once gestation is confirmed, pregnant sows are moved to gestation barns for 116 days, and one week prior to farrowing are transferred to maternity barns for a 23-day lactation period. On average, 12.5 live piglets are born per litter, with a mortality rate of 16%, meaning that 10.5 piglets are weaned at the end of the phase.

Piglets are weaned with an average weight of 7.37 kg, then transferred to nursery barns to consume pre-starter feed for around 26 days until they reach an average weight of 19.28 kg. Subsequently, the developing pigs are moved to a starting area to receive a starter diet for 26 days until they reach an average weight of 35.45 kg. Ultimately, pigs are transferred to fattening barns for 78 days: during the initial 26 days, pigs are fed with a growing diet until they reach a weight of 55.78 kg. Over the subsequent 26 days, they are fed a developing diet until a weight of 80.27 kg is reached. Lastly, pigs are provided a finishing diet for another 26 days until they reach market weight of 110 kg. All economic and financial data were obtained directly from the evaluated farm accounting sheets. An approval for field site access was not required since no field tests were carried out. Only data provided by the pork producers was gathered.

Ethics statement

The Ethics Committee of the Universidad Autónoma del Estado de México (México) approved the research protocol and information gathering tools. Prior to participation, the owner of the farm that conformed the analyzed RPPU150 was provided information about the overall aim and expected outcome of the present research. Due to the nature of the study, no animals were used to obtain data.

The model

A Stochastic simulation approach was carried out to evaluate the economic and financial viability of a representative medium-scale pig farm, using an empiric distribution [16], used by [13, 14] to evaluate Mexican pig farms and small-scale dairy systems, respectively. ~~using Zavala-Pineda et al. (2012) and Posadas-Domínguez et al. (2016) probability distributions.~~ Historical data for the probability distributions were taken from the [17]~~SIACON (2022)~~ database, that comprised 42 years of information (1981–2022). The studied model included data pertaining to the Mexican government to represent the national macroeconomic variables, while data from the International Monetary Fund -(IMF) and the United States Department of Agriculture (USDA) were used for the international macroeconomic variables.

According to [16], Empirical multivariate probability distributions (MVE) were estimated for each variable of the analyzed production unit. Historical data from the past ten years were used to estimate the MVEs. The MVEs were then used to simulate stochastic projections of the variables for the next five years with 500 iterations using the software [18].

In order to estimate the parameters for a Multivariate Empircal (MVE) distribution, the following steps were conducted: 1) Random and non-random components estimation for each stochastic variable (X^_it=a^+b^*Trend+c^Z_t) were separated; 2) The random component of each stochastic variable (e^_it=X_it-X^_it). were calculated; 3) Residuals (e^_it) were converted to relative deviates about their respective deterministic components; 4) The relative deviates (D_it=e^_it/X^_it) were sorted and pseudo-minimums and pseudo-maximums created for each random variable; 5) A probability of $P_{min} = Minimum\ S_{it} * 1.000001$ to $P_{max} = maximum * S_{it} * 1.000001$ with $S_{it} =$

Formatted: Indent: First line: 0.49"
Formatted: English (United States)
Formatted: English (United States), Not Strikethrough
Formatted: English (United States)
Formatted: English (United States), Strikethrough
Formatted: English (United States)
Formatted: English (United States)
Formatted: English (United States), Not Highlight
Formatted: Not Highlight
Formatted: English (United States), Not Highlight
Formatted: English (United States)
Formatted: Not Highlight
Formatted: English (United States)
Formatted: English (United States), Not Highlight
Formatted: Not Highlight
Formatted: English (United States)
Formatted: English (United States), Not Highlight
Formatted: English (United States)
Formatted: Font: 10 pt, Font color: Auto
Formatted: Font: 10 pt, Font color: Auto
Formatted: Font: 10 pt, Font color: Auto
Formatted: Font: 10 pt, Font color: Auto
Formatted: Font: 10 pt, Font color: Auto
Formatted: Font: 10 pt, Font color: Auto
Formatted: Font: 10 pt, Font color: Auto
Formatted: Font: 10 pt, Font color: Auto
Formatted: Font: 10 pt, Font color: Auto
Formatted: Font: 10 pt, Font color: Auto
Formatted: Font: 10 pt, Font color: Auto
Formatted: Font: 10 pt, Font color: Auto
Formatted: Font: 10 pt, Font color: Auto
Formatted: Font: 10 pt, Font color: Auto
Formatted: Font: 10 pt, Font color: Auto

$Sorted[D_{it} from \min to\ max]$ was assigned to each of the sorted deviates; 6) Calculate the M x M intra-temporal correlation matrix for the M random variables; and 7) The intertemporal correlation coefficients were calculated for the random variables. The seventh step completes the parameter estimation for a MVE distribution.

The complete MVE probability distribution was simulated in excel using Risk. The following steps were carried out: 1) A sample of Independent Standard Normal Deviates (ISND) was generated. ISND=Risknormal(0,1); 2) To simulate a MVE distribution the ISNDs within each year of the simulation period (k = 1, 2, ... , K) were correlated by multiplying the factored correlation matrix (R_{ij}) and eight of the values in the ISND vector (CSND_i=R_ij*ISND_i); 3) The inter-temporal correlation of the random variables was captured; 4) Equation Adjusted Correlated Standard Normal Deviates (ACSNDs) were transformed to Correlated Uniform Deviates (CUD). CUD_i=normsdist(ACSND_i); 5) CUD_is were used to simulate random deviates for the empirical distribution of each variable X_i; and 6) The correlated fractional deviates were applied to their respective projected means making any needed adjustments for heteroscedasticity. $\tilde{X}_i = \hat{X}_i * (1 + CFD_i * E_i$, with correlated fractional deviates (CFD) and expansion factor (E_i).

The model projected economic and financial viability and provided information on trends for macroeconomic variables. In order to reduce estimation errors, trends in regional variables were estimated for the period of 2022-2026. The model operates annually at a strategic level and generates financial pro-forma reports that contain results used to calculate key output variables such as net incomes in cash, final cash reserves, change in net real capital, net present value (NPV) and rate of return over assets (RRA), which can inform decision making at both the farm and policy levels. The said financial reports are generated from functional equations that link pig production, sales, production and purchase of inputs, capital operations, and consumption and financing operations.

Stochastic variables of the model

Formatted: Font: 10 pt, Font color: Auto

Formatted: Font: 10 pt, Font color: Auto

Formatted: Font: 10 pt, Font color: Auto

Formatted: Font: 10 pt, Font color: Auto

Formatted: Font: 10 pt, Font color: Auto

Formatted: English (United States)

Formatted: English (United States), Not Highlight

Formatted: English (United States)

Formatted: English (United States), Not Highlight

Formatted: English (United States)

Formatted: English (United States), Not Highlight

Formatted: English (United States)

Formatted: English (United States), Subscript, Not Highlight

Formatted: English (United States), Subscript

Formatted: English (United States)

Formatted: English (United States), Not Highlight

Formatted: English (United States)

Formatted: English (United States), Subscript, Not Highlight

Formatted: English (United States), Not Highlight

Formatted: English (United States)

Formatted: English (United States), Subscript

Formatted: English (United States)

Formatted: English (United States)

Formatted: English (United States), Subscript

Formatted: English (United States)

Formatted: English (United States), Not Highlight

Formatted: English (United States)

Formatted: Indent: First line: 0.49"

The inputs prices and yield of crops in the RPPU150 were obtained via meetings with producers and direct interviews. A Multivariate Empirical Distribution (MVE) was used to simulate the cost of crops and pigs. The probability distribution parameters were estimated with the Latin hypercube method, used as a sampling procedure for the simulation of pseudo-random numbers.

The use of such method ensures that the coefficient of variation (CV) and the mean for the simulated random variables are equal to the CV and the mean for the historic variables.

The stochastic values from the probability distributions are used in accounting equations to calculate production, receipts, costs, cash flows, and balance sheet variables for the project. Stochastic values sampled from the probability distributions make the financial statement variables stochastic, which are iterated 500 times using random values for the risky variables, the obtained information is used to estimate empirical probability distributions for the Key Output Variables (KOVs) such as present value of ending net worth, net present value, annual cash flows, amongst others. Providing forecasted KOVs that are used by decision makers [19, 20].

Economic and financial viability indicators

The ~~indicators~~ analysed indicators were total income (TI), total costs (TC), net income in cash (NIC), reserves in cash (RIC), NPV, RRA and cost to benefit ratio (C/B).

Model assumptions

The analysis took into account the following assumptions: (1) scale of production; (2) productivity; (3) farm infrastructure capacity and utilized; (4) technical coefficients are held constant during the planning horizon 2022-2026; (5) the technological level was also held unaltered; and (6) discount rate was established at 12%.

Economic values used were transformed in USD with a currency rate of 19.4143:1 USD:MXN according to Banco de Mexico (December 30, 2022).

Results and discussion

Formatted: Indent: First line: 0.49"

Formatted: Not Highlight

Formatted: English (United States), Not Highlight

Formatted: English (United States)

Formatted: English (United States), Ligatures: Standard + Contextual

Formatted: Line spacing: Multiple 1.08 li

Formatted: English (United States)

Formatted: Indent: First line: 0.49"

Formatted: Indent: First line: 0.49"

Table 1 shows the economic viability with a noticeable increase in all accounting entries. Gross income grew by 27.93% within the planning horizon, while expenses had a 16.09% reduction, this allowed for a 36% average annual growth of net income. The probability of falling into negative indicators was considerably reduced, with the cash flow deficit dropping from 40% in 2022 to 18% in 2026; the probability of refinancing decreased by 16 percentage points, and the probability of a return on assets less than zero dropped from 58% in 2022 to 13% in 2026.

Table 1. Economic viability of a RPPU150 (Thousands USD).

	2021	2022	2023	2024	2025	2026
Gross income	869.05	911.74	960.46	1,015.28	1,058.45	1,111.82
Total expenditure	849.98	889.93	903.38	926.69	958.39	986.76
Net income	19.07	21.81	57.08	88.59	100.07	125.06
Return on assets (%)	3.36	1.50	6.59	10.21	10.24	11.34
P(Cash Flow deficit) (%)	-	40.00	34.00	24.80	24.00	18.00
P(Refinancing) (%)	-	20.60	17.80	10.80	6.00	4.60
P(Return on assets < 0) (%)	-	58.00	46.00	32.20	21.20	13.00

P=Probability.

Net cash income performance within the planning horizon, according to the risk evaluation, showed that it is possible to fall in bankruptcy (5th percentile). However, 2026 economic panorama is a viable one (Fig 1).

Formatted: Indent: First line: 0.49"
Formatted Table
Formatted: Centered
Formatted: English (United States)
Formatted: Centered
Formatted: English (United States)
Formatted: Centered
Formatted: English (United States)
Formatted: Centered
Formatted: English (United States)
Formatted: Centered
Formatted: English (United States)
Formatted: Centered
Formatted: English (United States)
Formatted: Centered
Formatted: English (United States)
Formatted: Indent: First line: 0.49"

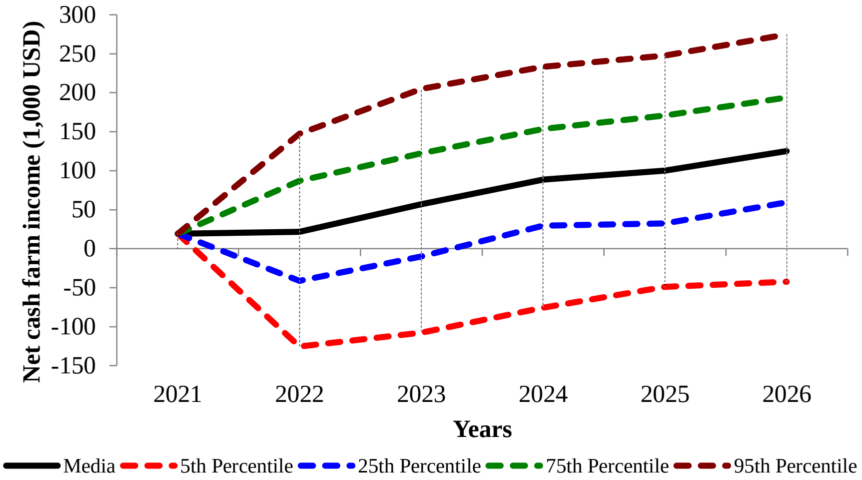

Figure 1. Net cash farm income performance according to percentile and risk simulation.

The cost structure was mainly composed of feed (88.17% of total), followed by ~~labour~~labor costs (5.79%), breeding costs (3.46%), and fuel costs (1.26%). Other concepts such as maintenance, veterinary service expenses, indirect expenses, transportation, and land costs, accounted for 0.79%, 0.47%, 0.03%, 0.02%, and 0.005% of the total costs, respectively.

In the evaluated RPPU150, there is a null probability of obtaining a negative Net Present Value (NPV). The NPV in 2026 finished in 46.9964 thousand USD. The Internal Rate of Return (IRR) was 11.06%. The Cost/Benefit ratio was 0.98 and the Real Rate of Return was 0.07%.

Around 1.7 million tons (MT) of pork were produced in Mexico in 2021 [17]~~(SIACON, 2022)~~, and the State of Mexico contributed with just over 22 thousand tons. Specifically, the said country had an average annual growth rate of 0.74, while the State of Mexico had a growth rate of -3.32 from 1980 to 2021.

In Mexico, the pig farming sector can be described in three historical and conjunctural periods [21] ~~(Bobadilla et al., 2010): ;~~ an initial period of growth driven by the structural policies established in the second half of the 1970s; a second period of crisis in the sector that began in 1985 and ended in 1991; and a third period starting from 1991 with a growing trend until 2021, mainly driven by the consolidation of corporate pig farming systems.

Formatted: English (United States)

Formatted: Indent: First line: 0.49"

Formatted: English (United States)

Formatted: English (United States)

Formatted: English (United States)

Formatted: English (United States)

The macroeconomic and sectoral policies adopted by Mexico in the last two decades has led to a change in the production structure of pig farming at the national and local level. While the State of Mexico is in the Central East region of Mexico, where growth and dynamism had positive effects during 1994 to 2012, the average annual growth rate for the said state was -1.31%.

One of the main effects observed in the Mexican pig industry was the impact of trade liberalization on the sector, mainly due to the competitive advantages of the United States over Mexico [22]~~(Tinoco, 2004)~~. The difference between harm to producers and the benefit to consumers, namely $14.0 billion USD, remained in the hands of pork importers and traders, as in Mexico, among other causes, an asymmetric price transmission exists due to an oligopolistic market where retailers tend to quickly pass on price increases to consumers, while delaying the decrease in prices when they have decreased for the producer [23] ~~(Gómez-Tenorio et al., 2020).~~. Such is the dependence on the external market that a 15% and 21% currency depreciation of the Mexican Peso would only imply an increase in national production of 0.3% and 0.4%, respectively [24] ~~(Almazan-Figueroa et al., 2018)~~.

The transition in the pig farming structure in the State of Mexico is mainly derived from technological progress, defined as feed conversion, and to a lesser extent, the price of feed and the price of pork, with average elasticity values of 1.174, -0.039, and -0.099, respectively [25]~~(Rebollar-Rebollar et al., 2014)~~.

In this regard, [26] ~~Rebollar-Rebollar et al. (2007)~~ determined that the best profit for pig farmers was obtained by producing animals with a range in live weight between 66 and 162 kg. However, the production of heavier carcasses did not necessarily imply a higher profit [25] ~~(Rebollar-Rebollar et al., 2014)~~. The latter authors also found that the Technical Optimum Level (TOL) and Economic Optimum Level (EOL) for pork carcass weight were 94.47 kg and 90.96 kg, respectively, with profits of USD$ 162.1 and USD$ 173.0. In the case of secondary cuts, TOL and EOL were obtained with respective carcass weights of 85.4 kg and 85.3 kg, with profits of USD$ 236.7 and USD$ 236.9, while for tertiary cuts, 82.38 kg (TOL) and 82.26 kg (EOL) were equivalent to profits of USD$ 217.6 and USD$ 217.9.

The State of Mexico used to be one of the main pork producing states. Unlike national pork production, that recovered from 1991, the aforesaid state’s pork industry was not able to do so, mainly due to the closure of a large part of its production units. While pork production at the national level grew

an average annual rate of 2.63% from 1990 to 2021, it decreased by 1.20% in the State of Mexico. The observed trend in the production volume of pork in Mexico support global projections that estimate an increase of pork mainly in low and middle-income countries [27] ~~(OECD/FAO 2022)~~, but the outlook is not the same for the pork industry in the State of Mexico. In this context, the estimation of financial projections has become a useful tool for assessing economic and financial viability of livestock systems. However, models must consider the conditions which prevail in the studied regions in order to reduce variability [28]~~(Zhang y Wang 2020)~~.

While the State of Mexico is a large consumer of pork, much of the said product is not produced in the state. One of the most significant changes from 2000 to 2019 was the concentration and commercialization of 21% of the slaughterhouses within the state. Additionally, 17 distribution centres and 24 slaughterhouses were located in the Central region of Mexico, representing 90% of the national traded volume [29]~~(Callejas-Juarez et al., 2020)~~.

Since 2006, a significant increase in the analysis of data pertaining to the pork sector has driven the development of models to assist decision-making [30] ~~(van Klompenburg and Kassahun, 2022)~~. The focus of these models is mainly related to diseases, DNA analysis, and feeding strategies.

Several studies have been conducted to measure the competitiveness of the pork industry at both the national and State levels in Mexico. By the end of the 2000s, [6]~~Hernandez-Martinez et al. (2008)~~ reported ~~favourable~~favorable private profitability indicators in small, medium, and large-scale pig farms in the State of Mexico. Meanwhile, [7] ~~Bobadilla-Soto et al. (2013)~~ reported private gains in the sale of weaned piglets in small-scale pig farms (5 to 100 breeding sows). However, ~~non of~~none of the aforesaid studies included a risk analysis component like the present study.

In Brazil, [31]~~Ali et al. (2018)~~ applied a stochastic model in a typical full-cycle farm (1500 sows) to evaluate the impact on private and social profits of using locally produced co-products in finishing diets, finding a high coefficient of variation (75-87%) in profits due to volatility in prices and variations in pig biological parameters. These results demonstrate the importance of considering risk factors in strategic planning for livestock businesses.

Formatted: English (United States)

Formatted: English (United States)

Formatted: English (United States)

Formatted: English (United States)

Formatted: English (United States)

Formatted: English (United States)

Formatted: English (United States)

Formatted: English (United States)

Formatted: English (United States)

Formatted: English (United States)

Formatted: English (United States)

On the other hand, [32]Calderón Díaz et al. (2019) generated a predictive model to evaluate the effect of making changes in the production system (increasing housing area to reach a higher market weight vs. feeding finishing feed at an earlier age), finding that the modification of feeding systems is the best alternative to increase farm income with a reduced investment. In our study, feeding represented 88% of the total cost structure.

The models of [26, 32, 33] Calderón Díaz et al. (2019), Rebollar-Rebollar et al. (2007), and Leen et al. (2018) allow for a clear visualisationvisualization, in economic and biological terms, which option offers greater productivity. [34] Davoudkhani et al. (2020) reported that optimizing strategies related to feeding and distribution can increase gross income per pig by up to 10%. All the aforesaid models developed to estimate profitability do not consider micro and macroeconomic variables, and are focused on biological components, as well as the price of some inputs for livestock feed.

The results of [35]Jia et al. (2021) warn that with the increase in the price of feed and land, the advantages of large-scale pig farms gradually become prominent, thus, forcing small and medium-scale farms to close. Additionally, since pig farming is inevitably linked with the environment and productivity levels, environmental legislation could have a strong impact on the future development of the pork sector [27](OECD/FAO, 2022).

[33]Leen et al. (2018) highlight the importance of individual farm modelling, since the combination of curves with different risk factors, production conditions, and payment curves in the same projection, could result in an underestimation of the economic and financial indicators.

Under the current technological, productive and management conditions, considering the macromacroeconomic and microeconomic outlook presented over the period 2022 to 20206, the RPPU150 showed favourablefavorable economic and financial indicators. While the studied scenario was favorable in its mean value, the results showed that there is a risk (5th percentile) of obtaining negative net cash income. Therefore, it is necessary to further analyze the farm's management of production and economic variables. A new prospective study is required given that the present analysis was conducted with a 12% interest rate, making the production unit highly vulnerable in financial terms. According to OECD estimations, a significant pressure on livestock input prices will predominate in the 2020-2030 decade [27].

Formatted: English (United States)

Formatted: English (United States)

Formatted: English (United States)

Formatted: English (United States)

Formatted: English (United States)

Formatted: English (United States)

Formatted: English (United States)

Formatted: English (United States)

Formatted: English (United States)

Formatted: English (United States)

Formatted: English (United States)

Formatted: English (United States)

Formatted: English (United States)

Formatted: English (United States)

Formatted: English (United States)

Since in the analyzed pig production system, 88% of the cost structure was composed of feed, it is important to develop strategies in collaboration with regional agricultural producers that generate benefits for both economic sectors. The goal is not only to ensure livestock feed inputs but also to create short marketing circuits that boost the local economy. While intensive pig production allows for the acquisition of a large volume of inputs, for a production unit with 150 sows (RPPU150), such economies of scale are not an option. Additionally, the acquisition of inputs from distant regions would have social and environmental implications [36].

While [37] demonstrated that small-scale pig farms (100 to 300 sows) were not competitive at private prices, profits at efficient prices increased in comparison to private prices, presenting a comparative advantage in international markets and contributing to 55% of the currency savings for the country that produced the pigs. However, it is important to consider the proper management of the macroeconomic policy related to the Mexican peso/US dollar exchange rate and interest rate since it may impose an additional tax per kilogram of live weight pig produced in Mexico. In this regard, [8] mentioned that a subsidy to the supply of pork would increase production, domestic consumption, and imports by 0.07%, 0.006%, and 0.003%, respectively. The Net Social Value would increase by 0.006%. Since Mexico is an importer of pork, achieving efficiency and competitiveness in this sector is crucial. [9] emphasize the role of possible import tariffs on pork as a protective measure for the sector. The latter authors report that a 20% tariff would increase domestic production by 0.4% (4,774 tons), reduce imports by 18.9% (19,141 tons), decrease consumption by 0.06% (14,367 tons), and reduce the Net Social Value by -0.1% (-$309,050,545 USD)

Conclusions

~~Our results~~The present results ~~showed~~ evidenced a consistent economic and financial viability scenario for the evaluated pig farm, under the current conditions, and within the analyzed planning horizon ~~of the Scale farms under the current conditions in the horizon planning.~~ Net income increased by 555%, return on assets rose from 3.36% in 2022 to 11.34% in 2026, and the probability of decapitalization dropped from 58% to 13%, respectively, in the aforesaid periods. Similarly, the

Formatted: Not Highlight

Formatted: Not Highlight

Formatted: Not Highlight

Formatted: Not Highlight

Formatted: Not Highlight

Formatted: English (United States)

Formatted: Indent: First line: 0.49"

Formatted: Indent: First line: 0.49"

Formatted: English (United States)

Formatted: English (United States)

Formatted: English (United States)

Formatted: English (United States)

Formatted: English (United States)

probability of obtaining negative net income decreased from 40% in 2022 to 18% in 2026. The technological, productive, and economic management of the production unit allowed for a ~~favourable~~favorable scenario within the planning horizon. Even with the general favorable scenario~~those data~~, a~~there is the~~ risk (5th percentile) exists of falling into bankruptcy. It is important to determine the economic and financial performance within a ~~, yn an~~planning horizon, ~~, planning because~~since ~~this size of farms~~ the analyzed system represents ~~are~~ typical small-scale pig farm ~~common~~ in the State of Mexico, which ~~, and~~ represents~~s~~ 30% of the total pig inventory and 30% of pork production.

Acknowledgements

The authors wish to thank the pork producer that kindly provided the information for this study. ~~and the research project 6498/2022CIB of the Universidad Autónoma del Estado de México for the financing of this research.~~

References

1. SIAVI. 2023. Comercio Exterior. Gobierno de México. http://www.economia-snci.gob.mx/ Accessed: May 20, 2022.

2. Martínez Castañeda FE, Ávila Pérez F, Trujillo Ortega ME, Peñuelas Rivas CG. El hecho de nacer en una pocilga, no te da derecho de ser un cerdo. In: Martínez Castañeda, F.E., Herrera Tapia F. (eds) 2020. Aprendizajes y trayectorias del sector agroalimentario mexicano durante el TLCAN. ISBN: 978-92-9248-885-7. Instituto Interamericano de Cooperación para la Agricultura (IICA). Pp. 163-178

3. FAO. Portal lácteo. https://www.fao.org/dairy-production-products/production/production-systems/es/. Accessed January 13, 2022.

4. Camacho Castellanos CM, Vega González M. Primeros pasos para planificar la transición generacional en una granja que es una empresa familiar. https://www.3tres3.com/latam/articulos/primeros-pasos-para-planificar-transicion-generacional-en-una-granja_14944/#:~:text=Seg%C3%BAn%20la%20FAO%20m%C3%A1s%20del,y%2015%25%20a%20la%20tercera. Consulted March 21, 2023. Acceced: November 12, 2023.

Formatted: English (United States)

Formatted: English (United States)

Formatted: English (United States)

Formatted: English (United States)

Formatted: English (United States)

Formatted: English (United States)

Formatted: English (United States)

Formatted: English (United States)

Formatted: English (United States)

Formatted: English (United States)

Formatted: English (United States)

Formatted: English (United States)

Formatted: English (United States)

Formatted: Font: 18 pt, Bold, English (United States)

Formatted: Font: 18 pt, Bold

5. Rota A. Desarrollo de la ganadería en pequeña escala. Fondo Internacional de Desarrollo Agrícola. 2016. https://www.ifad.org/documents/38714170/40237450/Scaling+up+Results+in+Smallholder+Livestock+Development_s.pdf/f7a33c96-ade3-4ce4-90c3-62431cada563 Accessed 30 March 2022

6. Hernández-Martínez J, Rebollar-Rebollar S, Rojo-Rubio R, García-Salazar JA, Guzmán-Soria E, Martínez-Tinajero JJ, Díaz-Carreño MA. Private profitability of swine farms in the South of the State of Mexico. Universidad y Ciencia. 2008;24:117-124.

7. Bobadilla-Soto EE, Espinoza-Ortega A, Martínez-Castañeda FE. Swine piglet breeding farms competitiveness and profitability. Revista Mexicana de Ciencias Pecuarias. 2013;4:87-92.

8. Rebollar-Rebollar S, Velázquez HH, Gómez-Tenorio G, Posadas-Domínguez RR, Martínez-Castañeda FE. Efectos de la aplicación de subsidios al mercado porcino en México. Archivos de Zootecnia. 2020a;69:30-37. https://doi.org/10.21071/az.v69i265.5036

9. Rebollar-Rebollar S, Gómez-Tenorio G, Martínez-Castañeda FE. Efectos de un arancel Ad Valorem sobre importaciones de carne de cerdo en México. Agricultura, Sociedad y Desarrollo. 2020b;17:697-712. https://doi.org/10.22231/asyd.v17i4.1400

10. Sosa Urrutia ME, Martínez Castañeda FE. Espinosa García JA, Buendía Rodríguez G. Contribución del sector pecuario a la economía Mexicana. Un análisis desde la Matriz Insumo Producto. Revista Mexicana de Ciencias Pecuarias. 2017;8:31-41. https://doi.org/10.22319/rmcp.v8i1.4308

11. Jonasson E, Filipski M, Brooks J, Taylord EJ. Modeling the welfare impacts of agricultural policies in developing countries. Journal of Policy Modeling. 2014;36:63–82. https://doi.org/10.1016/j.jpolmod.2013.10.002

12. Nadal-Roig E, Lluís M, Plà-Aragonès LM, Pagès-Bernaus A, Albornoz VM. A two-stage stochastic model for pig production planning in vertically integrated production systems. Computers and Electronics in Agriculture. 2020:176:105615. https://doi.org/10.1016/j.compag.2020.105615

13. Zavala-Pineda MJ, Salas-González JM, Leos-Rodríguez JA, Sagarnaga-Villegas LM. The building of representative hog farm production and analysis of its economic viability during the period 2009-2018. Agrociencia. 2012;46:731-743.

14. Posadas-Domínguez RR, Callejas-Juárez N, Arriaga-Jordán CM, Martínez-Castañeda FE. Economic and financial viability of small-scale dairy systemsin central Mexico: economic scenario 2010–2018. Tropical Animal Health and Production. 2016;48:1667-1671. https://doi.org/10.1007/s11250-016-1141-3

15. Callejas Juárez N, Rebollar Rebollar S. Rentabilidad y competitividad del sistema vaca-becerro en México. In: Martínez Castañeda FE, Herrera Tapia F. (eds) 2020. Aprendizajes y trayectorias del sector agroalimentario mexicano durante el TLCAN. ISBN: 978-92-9248-885-7. Instituto Interamericano de Cooperación para la Agricultura (IICA). Pp. 121-140.

16. Richardson, J.W., S.L. Klose, and A.W. Gray. 2000. An applied procedure for estimating and simulating multivariate empirical (MVE) probability distributions in farm level risk assessment and policy analysis. J. Agric. Appl. Econ. 32(2):299-315.

17. SIACON. 2022. Sistema de Información agroalimentaria de Consulta. Secretaria de Agricultura y Desarrollo Rural. México. Database.

18. Simetar (2008), Simulation and Econometrics to Analyze Risk, Department of Agricultural Economics, Texas A&M University, College Station, TX.

19. Richardson, J. W. (2006). “Simulation for applied risk management.” Unnumbered staff report, Department of Agricultural Economics, Agricultural and Food Policy Center, Texas A&M University, College Station, Texas.

20. Richardson, J.W., Herbst, B.K., Outlaw, and Chope Hill II, R. 2007. Including Risk in Economic Feasibility Analyses: The Case of Ethanol Production in Texas. Journal of Agribusiness 25,2(Fall 2007):115S132

21. Bobadilla-Soto EE, Espinoza-Ortega A, Martínez-Castañeda FE. Dinámica de la producción porcina en México de 1980 a 2008. Revista Mexicana de Ciencias Pecuarias. 2010;1;251-268.

Formatted: Not Highlight

Formatted: Not Highlight

Formatted: Not Highlight

22. Tinoco JJL. La Porcicultura mexicana y el Tratado de Libre Comercio de América del Norte. Universidad Nacional Autónoma de México. 2004.

23. Gómez-Tenorio G, Rebollar-Rebollar S, Velázquez-Villalva HH. Impacto económico del libre comercio de carne de cerdo en la porcicultura mexicana. In: Martínez Castañeda, F.E., Herrera Tapia F. (eds) 2020. Aprendizajes y trayectorias del sector agroalimentario mexicano durante el TLCAN. ISBN: 978-92-9248-885-7. Instituto Interamericano de Cooperación para la Agricultura (IICA). Pp. 107-119.

24. Almazan-Figueroa O, Rebollar-Rebollar S, Velázquez-Villalva HH, Gómez-Tenorio G, Hernández-Martínez J. Effects of Mexican peso depreciation on pork market. Agronomía Mesoamericana. 2018;29:557-569. https://doi:10.15517/ma.v29i3.30105

25. Rebollar Rebollar A, Gómez Tenorio G, Hernández Martínez J, Rebollar-Rebollar S, González Razo FJ. Behaviour of supply and demand of regional pig carcasses in Mexico. Revista Mexicana de Ciencias Pecuarias. 2014;5:377-392.

26. Rebollar Rebollar S, Gómez Tenorio G, Hernández Matínez J, Rojo Rubio R, González Razo FJ, Avilés Nova F. Determinación del óptimo técnico y económico en una granja porcícola en Temascaltepec, Estado de México. Ciencia Ergo Sum. 2007;14:255-262.

27. OECD/FAO, OECD-FAO Agricultural Outlook 2022-2031, OECD Publishing, Paris, 2022.

28. Zhang F, Wang F. Prediction of pork supply via the calculation of pig population based on population prediction model. International Journal of Agricultural and Biological Engineering. 2020;13:208–217. https://doi.org/10.25165/j.ijabe.20201302.5303

29. Callejas-Juárez N, Martínez-Castañeda FE, Rebollar-Rebollar S. Estructura de mercado para cerdos vivos en México. REDES, Revista Hispana para el Análisis de Redes Sociales. 2020;31:116-123. https://doi.org/10.5565/rev/redes.888

30. van Klompenburg T, Kassahun A. (2022). Data-driven decision making in pig farming: A review of the literature. Livestock Science. 2022:261:104961. https://doi.org/10.1016/j.livsci.2022.104961

Formatted: English (United States)

31. Ali BM, Berentsen PBM, Bastiaansen JWM, Oude Lansink, A. A stochastic bio-economic pig farm model to assess the impact of innovations on farm performance. Animal. 2018;12:819–830. https://doi.org/10.1017/S1751731117002531

Almazan-Figueroa O, Rebollar-Rebollar S, Velázquez-Villalva HH, Gómez-Tenorio G, Hernández-Martínez J. Effects of Mexican peso depreciation on pork market. Agronomía Mesoamericana. 2018;29:557-569. https://doi:10.15517/ma.v29i3.30105

Bobadilla-Soto EE, Espinoza-Ortega A, Martínez-Castañeda FE. Dinámica de la producción porcina en México de 1980 a 2008. Revista Mexicana de Ciencias Pecuarias. 2010;1;251-268.

Bobadilla-Soto EE, Espinoza-Ortega A, Martínez-Castañeda FE. Swine piglet breeding farms competitiveness and profitability. Revista Mexicana de Ciencias Pecuarias. 2013;4:87-92.

Callejas Juárez N, Rebollar Rebollar S. Rentabilidad y competitividad del sistema vaca-becerro en México. In: Martínez Castañeda FE, Herrera Tapia F. (eds) 2020. Aprendizajes y trayectorias del sector agroalimentario mexicano durante el TLCAN. ISBN: 978-92-9248-885-7. Instituto Interamericano de Cooperación para la Agricultura (IICA). Pp. 121-140.

Callejas-Juárez N, Martínez-Castañeda FE, Rebollar-Rebollar S. Estructura de mercado para cerdos vivos en México. REDES, Revista Hispana para el Análisis de Redes Sociales. 2020;31:116-123. https://doi.org/10.5565/rev/redes.888

Camacho Castellanos CM, Vega González M. Primeros pasos para planificar la transición generacional en una granja que es una empresa familiar. https://www.3tres3.com/latam/articulos/primeros-pasos-para-planificar-transicion-generacional-en-una-granja_14944/#:~:text=Seg%C3%BAn%20la%20FAO%20m%C3%A1s%20del,y%2015%25%20a%20la%20tercera. Consulted March 21, 2023. Acceced: November 12, 2023.

Davoudkhani M, Mahé F, Dourmad JY, Gohin A, Darrigrand E, Garcia-Launay F. Economic optimization of feeding and shipping strategies in pig-fattening using an individual-based model. Agricultural Systems. 2020;184:102899. https://doi.org/10.1016/j.agsy.2020.102899

Formatted: English (United States)

Formatted: English (United States)

Formatted: English (United States)

32. Calderón Díaz JA, Shalloo L, Niemi JK, Kyriazakis I, McKeon M, McCutcheon G, Manzanilla EG. Description, evaluation, and validation of the Teagasc Pig Production Model. Journal of Animal Science. 2019;97:2803–2821. https://doi.org/10.1093/jas/skz163

33. Leen F, Van den Broeke A, Aluwé M, Lauwers L, Millet S, Van Meensel J. (2018). Stakeholder-driven modelling the impact of animal profile and market conditions on optimal delivery weight in growing-finishing pig production. Agricultural Systems. 2018;162:34–45.

34. Davoudkhani M, Mahé F, Dourmad JY, Gohin A, Darrigrand E, Garcia-Launay F. Economic optimization of feeding and shipping strategies in pig-fattening using an individual-based model. Agricultural Systems. 2020;184:102899. https://doi.org/10.1016/j.agsy.2020.102899

FAO. Portal lácteo. https://www.fao.org/dairy-production-products/production/production-systems/es/. Accessed January 13, 2022.

Gómez-Tenorio G, Rebollar-Rebollar S, Velázquez-Villalva HH. Impacto económico del libre comercio de carne de cerdo en la porcicultura mexicana. In: Martínez Castañeda, F.E., Herrera Tapia F. (eds) 2020. Aprendizajes y trayectorias del sector agroalimentario mexicano durante el TLCAN. ISBN: 978-92-9248-885-7. Instituto Interamericano de Cooperación para la Agricultura (IICA). Pp. 107-119.

Hernández-Martínez J, Rebollar-Rebollar S, Rojo-Rubio R, García-Salazar JA, Guzmán-Soria E, Martínez-Tinajero JJ, Díaz-Carreño MA. Private profitability of swine farms in the South of the State of Mexico. Universidad y Ciencia. 2008;24:117-124.

35. Jia Y, Zhang Z, He Z, Zh, P, Zhang Y, Sun T. Production Efficiency Prediction of Pig Breeding Industry by Optimized LSTM Computer Algorithm under Environmental Regulation. Scientific Programming. 2021;1-12. https://doi.org/10.1155/2021/3074167

Jonasson E, Filipski M, Brooks J, Taylord EJ. Modeling the welfare impacts of agricultural policies in developing countries. Journal of Policy Modeling. 2014;36:63–82. https://doi.org/10.1016/j.jpolmod.2013.10.002

Leen F, Van den Broeke A, Aluwé M, Lauwers L, Millet S, Van Meensel J. (2018). Stakeholder-driven modelling the impact of animal profile and market conditions on optimal delivery weight in growing-

Formatted: English (United States)

Formatted: English (United States)

Formatted: English (United States)

Formatted: English (United States)

finishing pig production. Agricultural Systems. 2018;162:34–45. https://doi.org/10.1016/j.agsy.2018.01.013

Martínez Castañeda FE, Ávila Pérez F, Trujillo Ortega ME, Peñuelas Rivas CG. El hecho de nacer en una pocilga, no te da derecho de ser un cerdo. In: Martínez Castañeda, F.E., Herrera Tapia F. (eds) 2020. Aprendizajes y trayectorias del sector agroalimentario mexicano durante el TLCAN. ISBN: 978-92-9248-885-7. Instituto Interamericano de Cooperación para la Agricultura (IICA). Pp. 163-178

Nadal-Roig E, Lluís M, Plà-Aragonès LM, Pagès-Bernaus A, Albornoz VM. A two-stage stochastic model for pig production planning in vertically integrated production systems. Computers and Electronics in Agriculture. 2020:176:105615. https://doi.org/10.1016/j.compag.2020.105615

OECD/FAO, OECD-FAO Agricultural Outlook 2022-2031, OECD Publishing, Paris, 2022.

Posadas-Domínguez RR, Callejas-Juárez N, Arriaga-Jordán CM, Martínez-Castañeda FE. Economic and financial viability of small-scale dairy systemsin central Mexico: economic scenario 2010–2018. Tropical Animal Health and Production. 2016;48:1667-1671. https://doi.org/10.1007/s11250-016-1141-3

Rebollar Rebollar S, Gómez Tenorio G, Hernández Matínez J, Rojo Rubio R, González Razo FJ, Avilés Nova F. Determinación del óptimo técnico y económico en una granja porcícola en Temascaltepec, Estado de México. Ciencia Ergo Sum. 2007;14:255-262.

Rebollar Rebollar A, Gómez Tenorio G, Hernández Martínez J, Rebollar-Rebollar S, González Razo FJ. Behaviour of supply and demand of regional pig carcasses in Mexico. Revista Mexicana de Ciencias Pecuarias. 2014;5:377-392.

Rebollar-Rebollar S, Velázquez HH, Gómez-Tenorio G, Posadas-Domínguez RR, Martínez-Castañeda FE. Efectos de la aplicación de subsidios al mercado porcino en México. Archivos de Zootecnia. 2020a;69:30-37. https://doi.org/10.21071/az.v69i265.5036

Rebollar-Rebollar S, Gómez-Tenorio G, Martínez-Castañeda FE. Efectos de un arancel Ad Valorem sobre importaciones de carne de cerdo en México. Agricultura, Sociedad y Desarrollo. 2020b;17:697-712.

Formatted: English (United States)

Formatted: English (United States)

Formatted: English (United States)

Formatted: English (United States)

Rota A. Desarrollo de la ganadería en pequeña escala. Fondo Internacional de Desarrollo Agrícola. 2016. https://www.ifad.org/documents/38714170/40237450/Scaling+up+Results+in+Smallholder+Livestock+Development_s.pdf/f7a33c96-ade3-4ce4-90c3-62431cada563 Accessed 30 March 2022

SIACON. 2022. Sistema de Información agroalimentaria de Consulta. Secretaria de Agricultura y Desarrollo Rural. México. Database.

SIAVI. 2023. Comercio Exterior. Gobierno de México. http://www.economia-snci.gob.mx/ Acceced: May 20, 2022.

Sosa Urrutia ME, Martínez Castañeda FE. Espinosa García JA, Buendía Rodríguez G. Contribución del sector pecuario a la economía Mexicana. Un análisis desde la Matriz Insumo Producto. Revista Mexicana de Ciencias Pecuarias. 2017;8:31-41.

Tinoco JJL. La Porcicultura mexicana y el Tratado de Libre Comercio de América del Norte. Universidad Nacional Autónoma de México. 2004.

van Klompenburg T, Kassahun A. (2022). Data-driven decision making in pig farming: A review of the literature. Livestock Science. 2022:261:104961. https://doi.org/10.1016/j.livsci.2022.104961

Zavala-Pineda MJ, Salas-González JM, Leos-Rodríguez JA, Sagarnaga-Villegas LM. The building of representative hog farm production and analysis of its economic viability during the period 2009-2018. Agrociencia. 2012;46:731-743.

Zhang F, Wang F. Prediction of pork supply via the calculation of pig population based on population prediction model. International Journal of Agricultural and Biological Engineering. 2020;13:208–217.

36. Villavicencio- Gutiérrez MR, Rogers- Montoya NA, Martínez- Campos AR, Gómez- Tenorio G, Martínez- Castañeda FE. The environmental performance of different pork production scenarios: a life cycle assessment study. Tropical Animal Health and Production. 2021;54:44 https://doi.org/10.1007/s11250-022-03045-6

Formatted: English (United States)

Formatted: English (United States)

Formatted: English (United States)

Formatted: English (United States)

Formatted: Line spacing: Double

Formatted: English (United States)

Formatted: English (United States)

Formatted: English (United States)

Formatted: English (United States)

37. Barrón-Aguilar JF, García-Mata R, Mora-Flores JS, López-Díaz S, Pró-Martínez, García-Sánchez R. 2000. Competitiveness and the effect of economic policy on hog production in 13 farms in the state of Michoacán, 1995. Agrociencia. 34:369-377.

Formatted: Line spacing: Double

Formatted: English (United States)

According to reviewers' suggestions the following changes were made to the original manuscript:

Title page:

Lines 3 to 5: Ampersand symbol was included.

Line 24: The statement "[&]These authors also contributed equally to this work" was included.

Introduction:

Line 41: number "1" was replaced by the word "one".

Material and Methods:

Line 96 to 98: The following statement was included "An approval for field site access was not required since no field tests were carried out. Only data provided by the pork producers was gathered."

Line 143: Table 1 is cell-based.

Regarding the reviewer question about the model used: "The authors state they used the probability distributions of Zavala-Pineda et al. (2012) and Posadas-Domínguez et al. The authors must clarify which probability distributions (triangular, Gaussian, etc....) they used and why they were adequate for the case study"

Indeed, the probability distributions of Zavala-Pineda et al. and Posadas-Domínguez et al. were used. However, it should be clarified that both latter studies employed an empirical distribution based on the model developed by Richardson, J.W., S.L. Klose, and A.W. Gray. 2000. An applied procedure for estimating and simulating multivariate empirical (MVE) probability distributions in farm level risk assessment and policy analysis. J. Agric. Appl. Econ. 32(2):299-315.

The following paragraph was included:

Line 110-113: According to [16], Empirical multivariate… …the next five years with 500 iterations using the software [18].

Regarding the reviewer question about the package used to perform the quantitative analysis:

The package used to make the estimations was SIMETAR©

Regarding the reviewer question: "It is not clear what exactly the authors are analyzing. I would expect a representative cash flow (a figure) representing all variables considered"

While a figure representing all considered variables was not included in the manuscript, the following paragraphs were included for a better understanding of the Materials and Methods section:

Line 114 to 124: The following paragraph was included "In order to estimate the parameters for a Multivariate Empircal (MVE) distribution… …. The seventh step completes the parameter estimation for a MVE distribution."

Line 125 to 135: The following paragraph was included "The complete MVE probability distribution was simulated… … fractional deviates (CFD) and expansion factor (Ei)."

Regarding the reviewer question: "What are the necessary investments and costs for the studied farm? What are the empirical data applicable to this case study?"

The following statement was included:

Line 93 to 94: “All economic and financial… …accounting sheets.”

Regarding the reviewer observation about the Stochastic Variables of the Model: “Again, the authors provide a too-short description of the methodological steps. How many simulations were performed? Please provide details and clarify all steps.”

The following paragraph was included:

Line 151 to 157: “The stochastic values from the probability distributions… … KOVs that are used by decision makers [19, 20].”

Results and Discussion

Regarding the reviewer question: “What are the contributions and the practical implications of this study? I expect a profound discussion on this matter in this section.”

The following paragraphs were included:

Line 273 to 279: “Since in the analyzed pig production system… … regions would have social and environmental implications [36].

Line 280 to 292: “While [37] demonstrated that small-scale pig farms… … Net Social Value by -0.1% (-$309,050,545 USD)”

Acknowledgements

Line 254: The statement “The authors wish to thank the pork producer that kindly provided the information for this study, and the research project 6498/2022CIB of the Universidad Autónoma del Estado de México for the financing of this research” was replaced by “The authors wish to thank the pork producer that kindly provided the information for this study”.

References

Following the journal’s submission guidelines, the citing style was changed to numbers in square brackets (e.g. [1], [2]) and references are listed in order of appearance.

The following citations and references are included in the manuscript to support the modifications made in the Materials and Methods section and in the discussion:

16. Richardson et al. (2000); **17.** Richardson et al. (2006); **18**. Richardson et al. (2007); **36**. Villavicencio-Guitíerrez et al. (2021); and **37**. Barrón-Aguilar et al. (1995).
