# Supplementary material for: Economic and financial viability of a pig farm in central semi-tropical Mexico: 2022–2026 prospective
Source: PLoS One. 2024 May 9;19(5):e0298897. doi: 10.1371/journal.pone.0298897 (PMC11081355; doi:10.1371/journal.pone.0298897)
Supplement: Attachment — Submitted filename: Response to reviewers.docx [file pone.0298897.s004.docx]

According to reviewers’ suggestions the following changes were made to the original manuscript:

**Title page:**

**Lines 3 to 5**: Ampersand symbol was included.

**Line 24**: The statement “^&^These authors also contributed equally to this work” was included.

**Introduction:**

**Line 41:** number “1” was replaced by the word “one”.

**Material and Methods:**

**Line 96 to 98**: The following statement was included “An approval for field site access was not required since no field tests were carried out. Only data provided by the pork producers was gathered.”

**Line 143:** Table 1 is cell-based.

**Regarding the reviewer question about the model used:** “The authors state they used the probability distributions of Zavala-Pineda et al. (2012) and Posadas-Domínguez et al. The authors must clarify which probability distributions (triangular, Gaussian, etc....) they used and why they were adequate for the case study”

Indeed, the probability distributions of Zavala-Pineda et al. and Posadas-Domínguez et al. were used. However, it should be clarified that both latter studies employed an empirical distribution based on the model developed by Richardson, J.W., S.L. Klose, and A.W. Gray. 2000. An applied procedure for estimating and simulating multivariate empirical (MVE) probability distributions in farm level risk assessment and policy analysis. J. Agric. Appl. Econ. 32(2):299-315.

The following paragraph was included:

**Line 110-113:** According to [16], Empirical multivariate… …the next five years with 500 iterations using the software [18].

**Regarding the reviewer question about the package used to perform the quantitative analysis:**

The package used to make the estimations was SIMETAR^©^

**Regarding the reviewer question: “**It is not clear what exactly the authors are analyzing. I would expect a representative cash flow (a figure) representing all variables considered”

While a figure representing all considered variables was not included in the manuscript, the following paragraphs were included for a better understanding of the Materials and Methods section:

**Line 114 to 124:** The following paragraph was included “In order to estimate the parameters for a Multivariate Empircal (MVE) distribution… …. The seventh step completes the parameter estimation for a MVE distribution.”

**Line 125 to 135:** The following paragraph was included “The complete MVE probability distribution was simulated… … fractional deviates (CFD) and expansion factor (Ei).”

**Regarding the reviewer question:** “What are the necessary investments and costs for the studied farm? What are the empirical data applicable to this case study?”

The following statement was included:

**Line 93 to 94**: “All economic and financial… …accounting sheets.”

**Regarding the reviewer observation about the Stochastic Variables of the Model:** “Again, the authors provide a too-short description of the methodological steps. How many simulations were performed? Please provide details and clarify all steps.”

The following paragraph was included:

**Line 151 to 157**: “The stochastic values from the probability distributions… … KOVs that are used by decision makers [19, 20].”

**Results and Discussion**

**Regarding the reviewer question: “**What are the contributions and the practical implications of this study? I expect a profound discussion on this matter in this section.”

The following paragraphs were included:

**Line 273 to 279:** “Since in the analyzed pig production system… … regions would have social and environmental implications [36].

**Line 280 to 292**: “While [37] demonstrated that small-scale pig farms… … Net Social Value by -0.1% (-$309,050,545 USD)”

**Acknowledgements**

**Line 254**: The statement “The authors wish to thank the pork producer that kindly provided the information for this study, and the research project 6498/2022CIB of the Universidad Autónoma del Estado de México for the financing of this research” was replaced by “The authors wish to thank the pork producer that kindly provided the information for this study”.

**References**

Following the journal’s submission guidelines, the citing style was changed to numbers in square brackets (e.g. [1], [2]) and references are listed in order of appearance.

The following citations and references are included in the manuscript to support the modifications made in the Materials and Methods section and in the discussion:

**16.** Richardson et al. (2000); **17.** Richardson et al. (2006); **18**. Richardson et al. (2007); **36**. Villavicencio-Guitíerrez et al. (2021); and **37**. Barrón-Aguilar et al. (1995).
