# Supplementary material for: Economic and financial viability of a pig farm in central semi-tropical Mexico: 2022–2026 prospective
Source: PLoS One. 2024 May 9;19(5):e0298897. doi: 10.1371/journal.pone.0298897 (PMC11081355; doi:10.1371/journal.pone.0298897)
Supplement: Attachment — Submitted filename: Rebuttal letter.docx [file pone.0298897.s006.docx]

| 1. Review reference list | The list of references was reviewed; no citations were removed; five citations and respective references were included in the manuscript to support the modifications made in the first revision.  Changes made in the reference section:  **Line 360:** Reference number 18  18. Simetar (2008), Simulation and Econometrics to Analyze Risk, Department of Agricultural Economics, Texas A&M University, College Station, TX,  was changed to  18. Richardson (2008), Simulation and Econometrics to Analyze Risk, Department of Agricultural Economics, Texas A&M University, College Station, TX.  **Line 409:** https://doi.org/10.1016/j.agsy.2018.01.013 was removed and placed in **line 402**, since it corresponds to the DOI of reference number 33.  33. Leen F, Van den Broeke A, Aluwé M, Lauwers L, Millet S, Van Meensel J. (2018). Stakeholder-driven modelling the impact of animal profile and market conditions on optimal delivery weight in growing-finishing pig production. Agricultural Systems. 2018;162:34–45. https://doi.org/10.1016/j.agsy.2018.01.013  It should also be clarified that in the previous rebuttal letter, the order in which the five added citations and references were reported was not correct.  16. Richardson et al. (2000); 18. Richardson et al. (2008); 19. Richardson et al. (2006); 36. Villavicencio-Guitíerrez et al. (2021); and 37. Barrón-Aguilar et al. (1995). |
| --- | --- |
| 2. Have the authors made all data underlying the findings in their manuscript fully available? | We apologize but we are not clear on what is being requested. We are sharing an Excel file that contains the input data and results obtained (output data from the software). We don't have our database available in a repository; however, we are willing to share the database upon reasonable request. |
| 3. Some recommendations made by the reviewer were not attended | We believe we have addressed all the comments made by the reviewer; however, we did not present the variables as a cash flow because we considered that the number of variables involved would result in a too large figure. In the first revision, we preferred to add additional paragraphs to the Materials and Methods section to clarify how the parameters used to make the estimations were obtained. |
|  |  |
| 4. In the current version there are revisions regarding grammar. A careful revision is strongly recommended. | **Line 34**: The word “favourable” was changed to “favorable”.  **Line 106**: The word “that” was changed to “which”.  **Line 114:** The word “Empircal” was changed to “Empirical”.  **Line 125-126**: The sentence “The complete MVE probability distribution was simulated in Excel using Risk.” Was changed to “The complete MVE probability distribution was simulated with the Microsoft Excel add-in tool @RISK.”.  **Line 149**: “coefficient of variation” was changed to “Coefficient of Variation”.  **Line 159**: “analysed” was changed to “analyzed”.  **Line 162:** “took into account” was changed to “considered”.  **Line 213-214:** “was obtained by producing animals” was changed “was obtained when they produced animals”.  **Line 222:** “that recovered from 1991” was changed to “which recovered since 1991”.  **Line 226**: “mainly” was changed to “primarily”.  **Line 232**: “ in” was changed to “within”.  **Line 234**: “centres” was changed to “centers”.  **Line 243:** “aforesaid” was changed to “latter”. |
|  |  |
